# Supplementary material for: CELF1 is a central node in post-transcriptional regulatory programmes underlying EMT
Source: Nat Commun. 2016 Nov 21;7:13362. doi: 10.1038/ncomms13362 (PMC5121338; doi:10.1038/ncomms13362)
Supplement: Supplementary Information — Supplementary Figures 1-12 and Supplementary Tables 1-3 [file ncomms13362-s1.pdf]

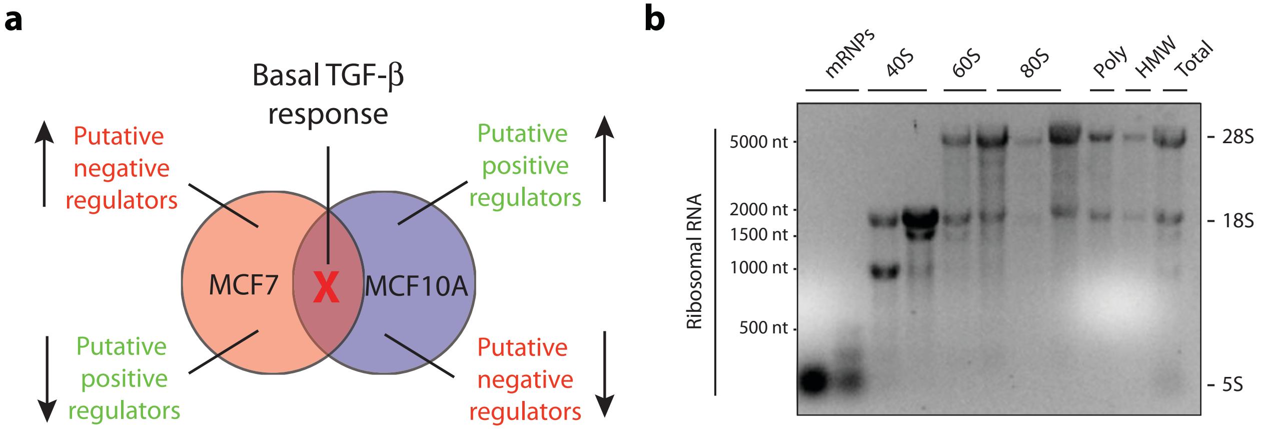

Supplementary Figure 1. Profiling and Isolation of Polysomal Fractions from MCF10A and MCF7 cells. (**a**) Rationale for polysomal profiling in both MCF10A cells, which are responsive to TGF-β, and MCF7 cells, which are not. The TGF-β signaling pathway is intact in both cell lines. (**b**) Representative ribosomal RNA profile of different fractions visualized by denaturing agarose gel to confirm fractionation fidelity. S = Svedberg unit; Poly = pooled polysomal fractions; HMW = high molecular weight fractions. Images is representative of a minimum of three individual experimental replicates.

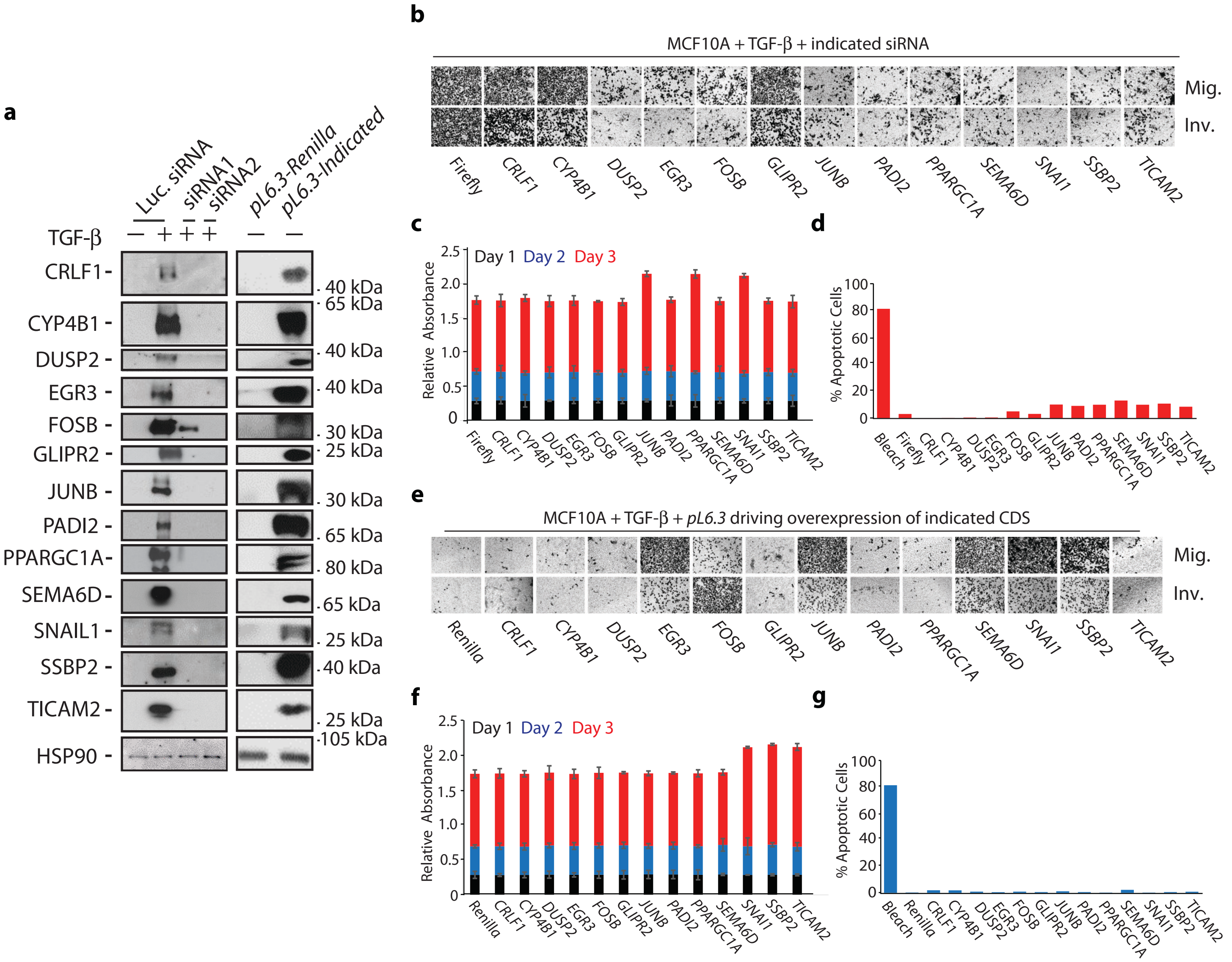

Supplementary Figure 2. Effective Knockdown and Overexpression of GRE-containing Transcripts Impacts MCF10A Migration and Invasion but has Largely Negligible Effects on Viability and Proliferation. (**a**) Representative analysis of protein levels in MCF10A cells transiently transfected with siRNAs targeting *Firefly* Luciferase or indicated gene products (*left*), or transduced with *pL6.3-Renilla* Luciferase or *pL6.3*-indicated genes (*right*). Each blot was probed for HSP90 to confirm equal loading - a representative blot is shown. (**b**) Representative crystal violet-stained images of migratory (*Mig.*) and invasive (*Inv.*) cells transfected with the indicated siRNAs. (**c**) Cell proliferation was evaluated via MTT assays over the course of three days in cells transfected with the indicated siRNAs. Absorbance of culture medium at 570 nM is shown. (**d**) Cell viability in cells transfected with the indicated siRNAs was assessed via Annexin V/PI staining. As a positive control, apoptosis/cell death was induced in non-manipulated cell by adding bleach to culture medium at a final concentration of 0.1% for 10 minutes. (**e**) Representative crystal violet-stained images of migratory (*Mig.*) and invasive (*Inv.*) cells transfected with the indicated overexpression constructs. (**f**) Cell proliferation was evaluated via MTT assays over the course of three days in cells transfected with the indicated overexpression constructs. Absorbance of culture medium at 570 nM is shown. (**g**) Cell viability in cells transfected with the indicated overexpression constructs was assessed via Annexin V/PI staining. As a positive control, apoptosis/cell death was induced in non-manipulated cell by adding bleach to culture medium at a final concentration of 0.1% for 10 minutes. All panels are representative of a minimum three independent experimental replicates. Error bars depict standard deviation of the mean (SD). Full scans of blots are shown in **Supplementary Figure 9**.

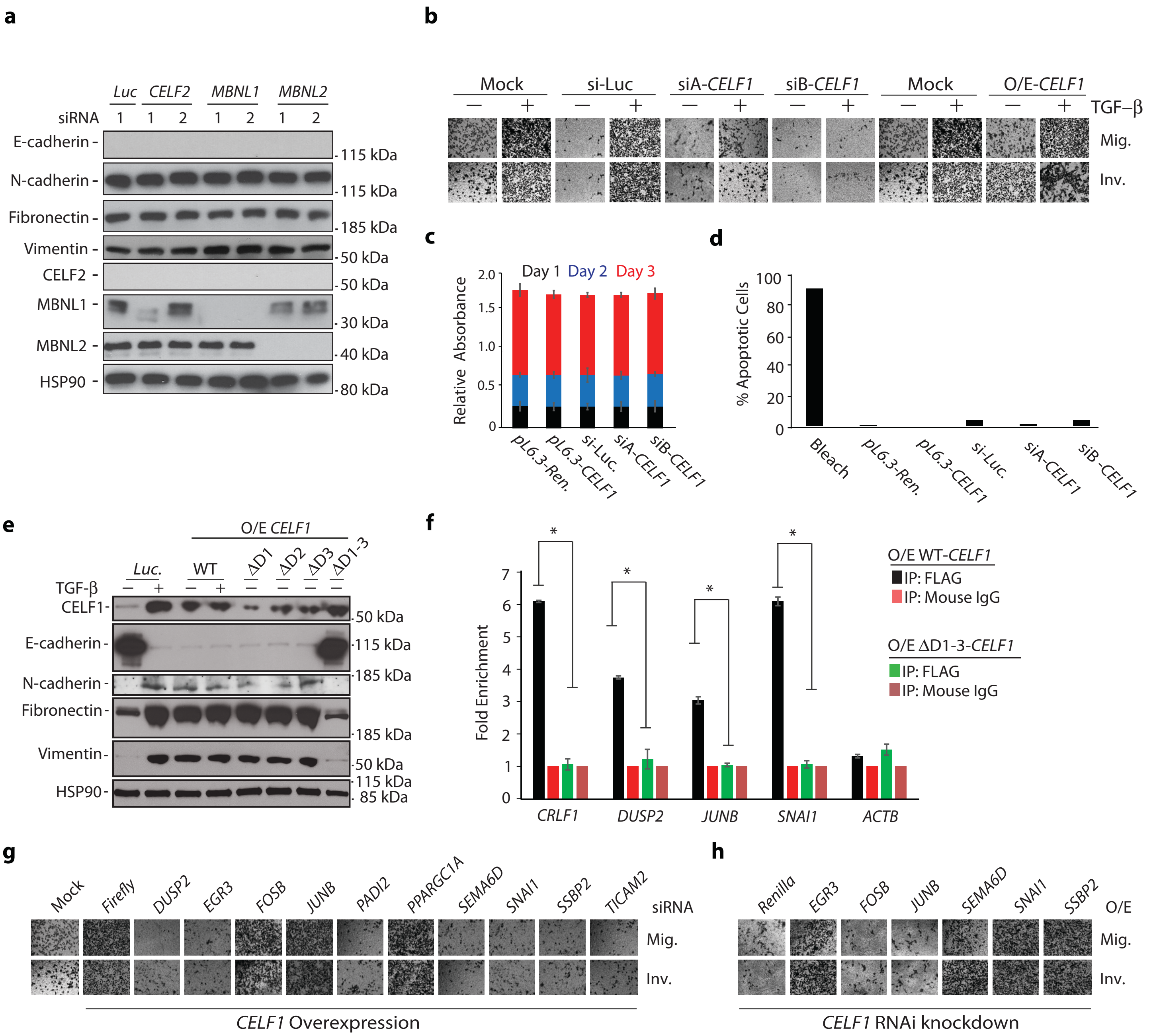

Supplementary Figure 3. CELF1 is Necessary for and Sufficient to Drive EMT in MCF10A cells, Sufficiency Requires CELF1 RNA-Binding Activity, and Genetic Ordering of *CELF1* and GRE-containing EMT Effectors. (**a**) RNAi-mediated silencing of *CELF2*, *MBNL1*, or *MBNL2* does not inhibit TGF-β induced EMT in MCF10A cells, as assessed by failure to prevent gain and loss of mesenchymal and epithelial cell markers, respectively. (**b**) *In vitro* migration (*Mig.*) and invasion (*Inv.*), (**c**) cell proliferation, and (**d**) viability was evaluated in indicated transfectants/transductants, respectively. (**e**) Immunoblot analysis of indicated epithelial and mesenchymal cell markers in untreated or TGF-β treated MCF10A cells transiently transfected with the indicated overexpression constructs. For the experiments depicted in (**a**) and (**e**), data depicts results from a single representative experiment in which extracts were loaded to gels that were processed in parallel. Each blot was probed for HSP90 to confirm equal loading - a representative blot is shown. (**f**) RNA crosslinking-immunoprecipitation/qRT-PCR of GRE-containing mRNAs from untreated MCF10A cells transfected with either wild-type (WT) or RNA-binding mutant (ΔD1-3) *CELF1* using anti-FLAG antibody or mouse IgG. *ACTB* is a non-GRE containing negative control. (**g** and **h**) Representative crystal violet-stained images of migratory (*Mig.*) and invasive (*Inv.*) cells transfected with the indicated siRNAs or overexpression constructs. All panels are representative of a minimum of three independent experimental replicates except for panels e and f, which are representative of two independent experiments. For immunoblots depicted, samples were derived from the same experiment and gels were processed in parallel. In panels *c* and *d*, error bars represent standard deviation (SD) of the mean. In panel *f*, error bars represent standard error (SEM) of the mean. * = pval ≤ 0.05 (Student's t-test). Full scans of blots are shown in **Supplementary Figure 10**.

Chaudhury_FigS4

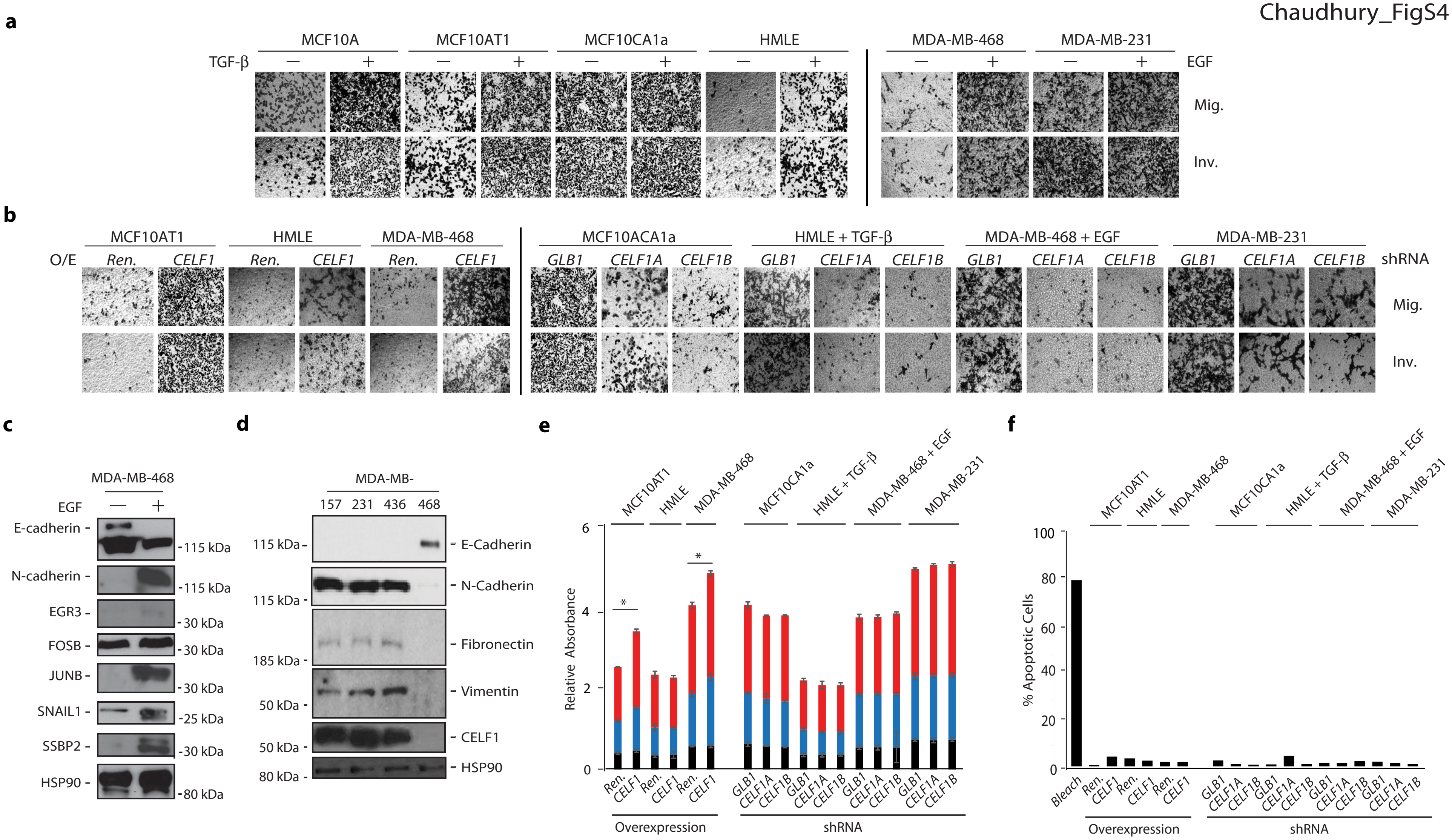

Supplementary Figure 4. CELF1 Basal Expression and Misexpression in a Panel of Breast Cancer Cell lines. (**a** and **b**) Representative crystal violet-stained images of migratory (*Mig.*) and invasive (*Inv.*) cells treated with the indicated EMT inducers **(a)** or transfected with the indicated *CELF1* overexpression or shRNA constructs. (**c**) Immunoblot analysis of proteins encoded by GRE-containing mRNAs in untreated and EGF-treated MDA-MB-468 cells. (**d**) Immunoblot analysis of epithelial markers, mesenchymal markers, and CELF1 in indicated cell types. Note that basal CELF1 protein expression correlates with the *in vivo* metastatic potential of each of the cell lines. For (**c**) and (**d**), samples were derived from the same experiment and gels were processed in parallel. Each blot was probed for HSP90 to confirm equal loadting - a represenative blot is shown. (**e**) The impact of misexpression of *CELF1* on proliferation of each of the indicated cell lines was assessed via MTT assay. (**f**) The impact of misexpression of *CELF1* on viability of each of the indicated cell lines was assesed via Annexin V/PI staining. In panels (**a**), (**b**), (**e**) and (**f**): *Ren.* = *Renilla* luciferase; *GLB1* = shRNA targeting β-galactosidase (*GLB1*). All panels are representative of a minimum of three individual experimental replicates. For immunoblots depicted, samples were derived from the same experiment and gels were processed in parallel. Error bars depict standard error (SEM) of the mean. * = pval ≤ 0.05 (Student's t-test). Full scans of blots are shown in Supplementary Figure 11.

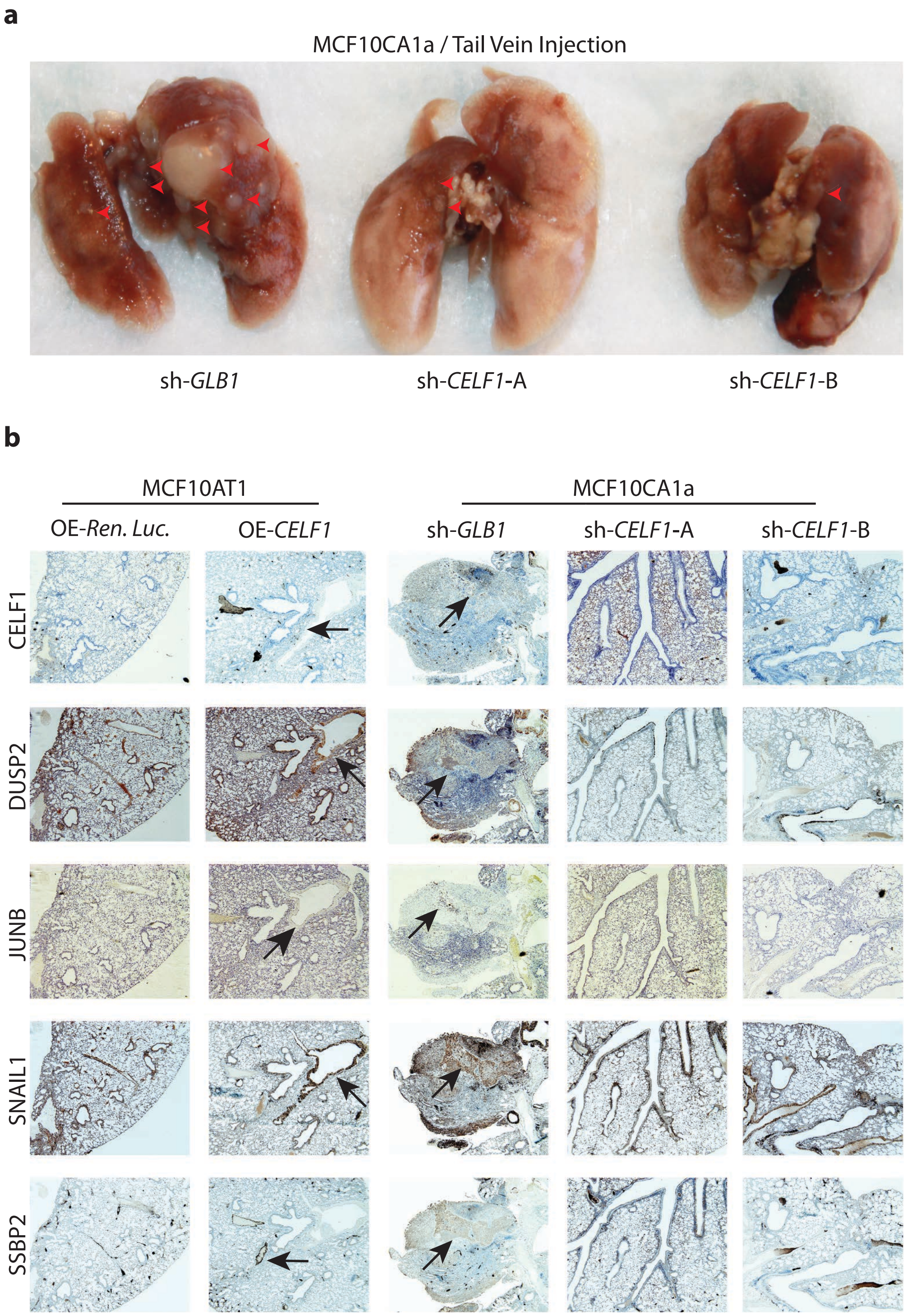

Supplementary Figure 5. Manipulation of *CELF1* Expression Impacts *In Vivo* Metastatic Colonization and Protein Expression of the GRE-containing mRNAs. (a) Representative images of gross anatomy of surgically excised and 10% neutral-buffered formalin fixed lungs used to count metastatic nodules (red arrow-heads). Images are representative of at least three individual experimental replicates. (b) Representative images of expression of CELF1 and indicated GRE-containing gene products in lungs from mice in Figure 6a and 6c. Black arrows represent micrometastasis. All images were obtained with 40x objective.

a

	Type	N	Minimum	25th Pctl	Median	75th Pctl	Maximum
Matched	Normal	29	0	1	20	60	85
	Tumor	29	1	40	70	90	100
Total	Normal	76	0	1	10	60	100
	Tumor	140	0	40	80	100	100

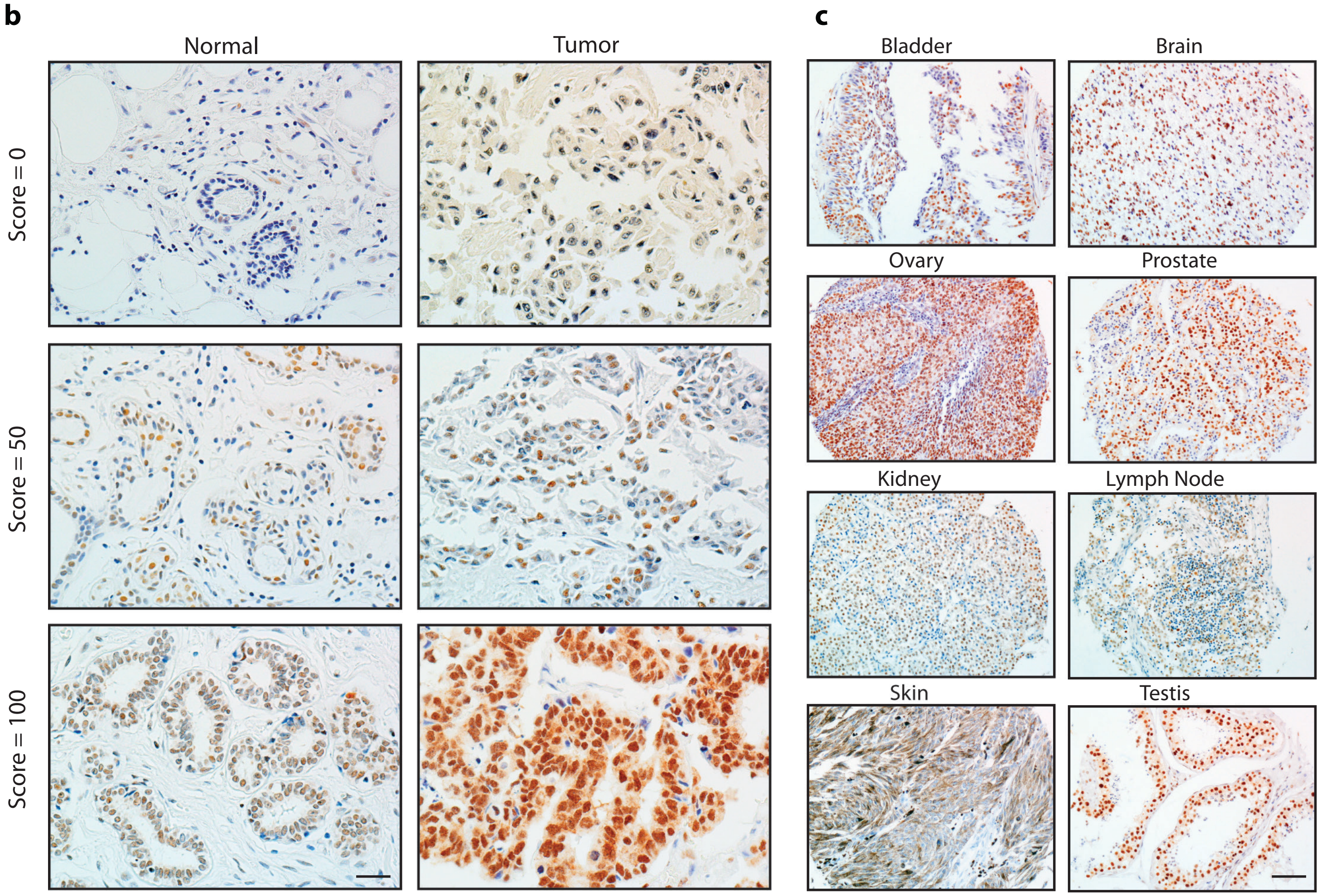

Supplementary Figure 6. Increased CELF1 Protein Expression in Human Cancers. (**a**) Comparison of CELF1 protein expression as assesed by IHC in human breast tumor and adjacent normal tissue speciments, both matched and non-matched. Shown are CELF1 histio scores that were calculated as percent staining (0-100). Pctl, percentile. (**b**) Representative images of human normal and breast cancer tumor tissues with CELF1 histio scores of 0, 50, and 100. (**c**) Representative images of positive CELF1 expression in indicated tumor tissues (bladder - 20%; brain - 20%; ovary - 10%; prostate - 5%; kidney - 10%; lymph nodes - 10%; skin - 13.3%; testis - 5% cases were positive). All images were obtained with 20X objective.

Supplementary Figure 7a

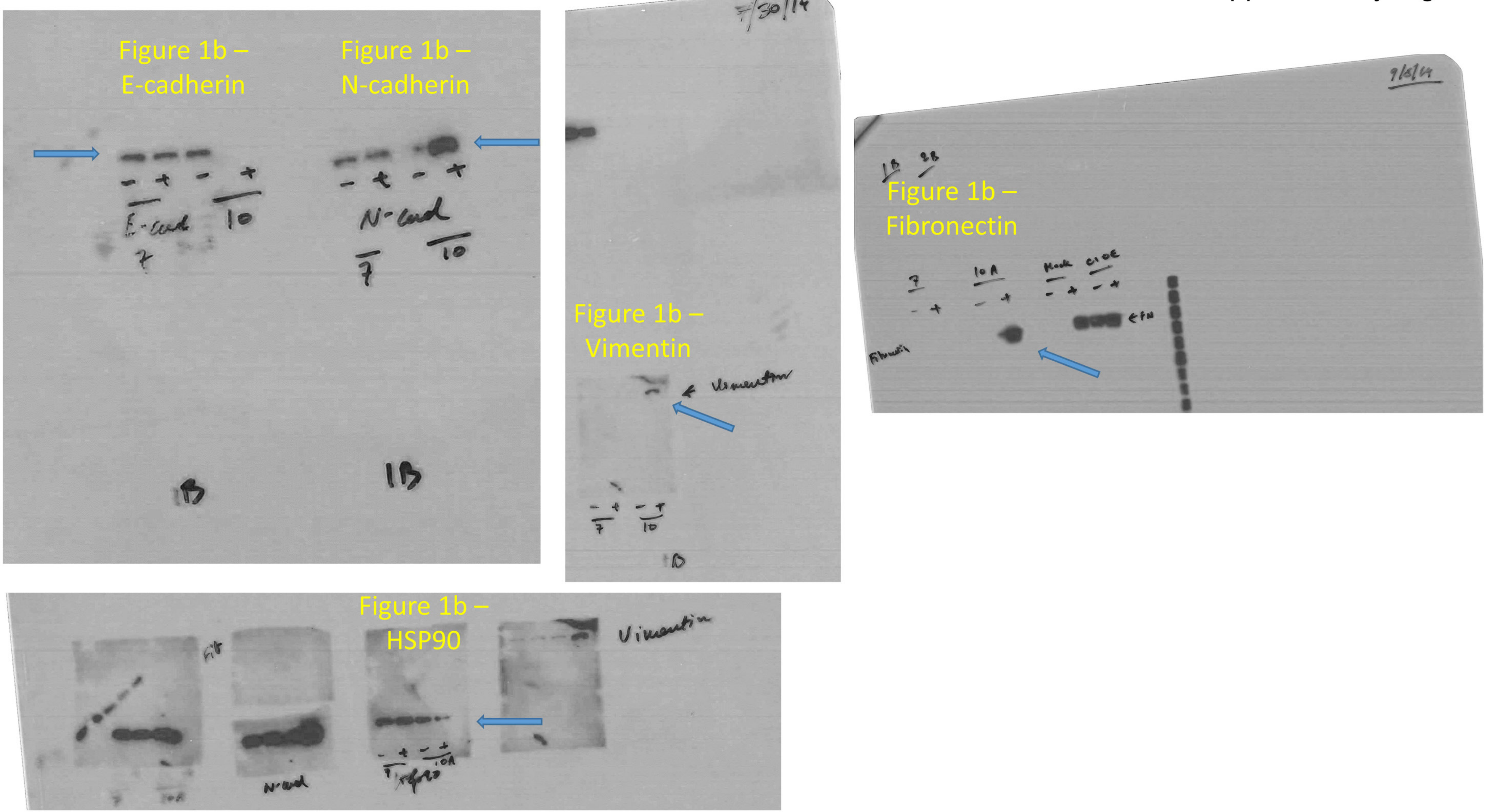

Figure 1d –
eIF3C

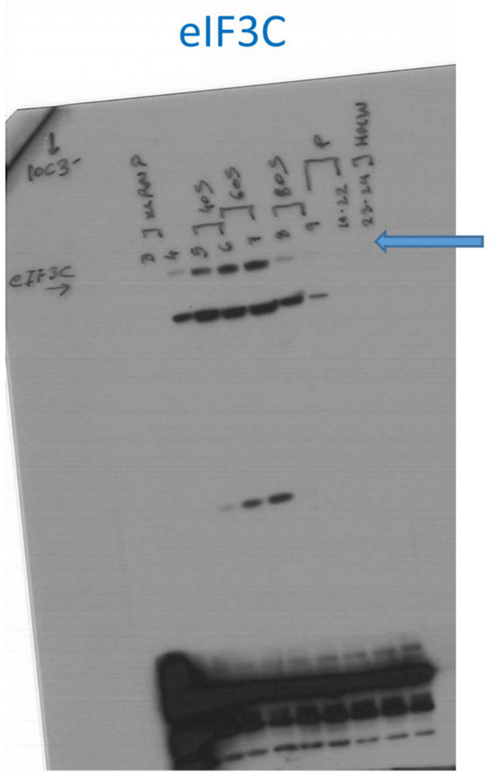

Figure 1d –
rPS6

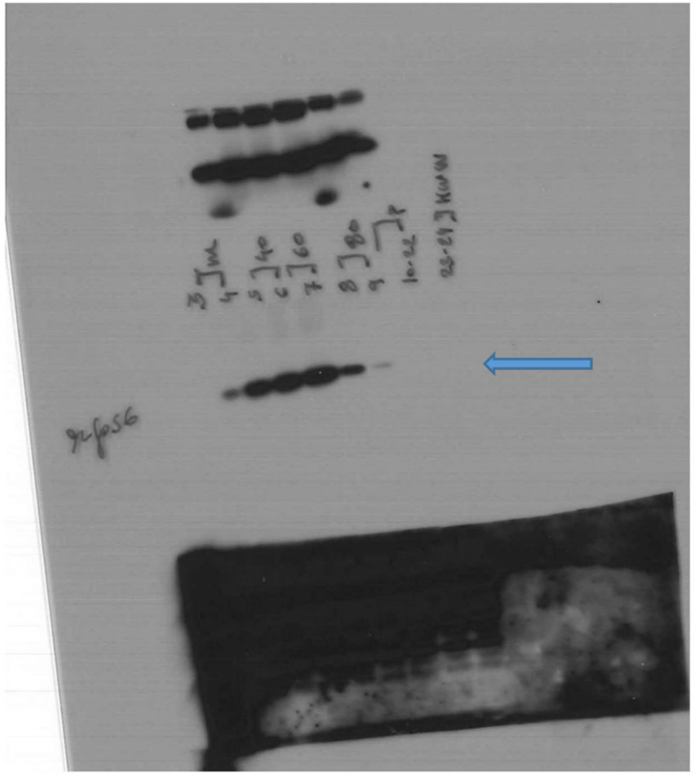

Figure 1d –
PABP

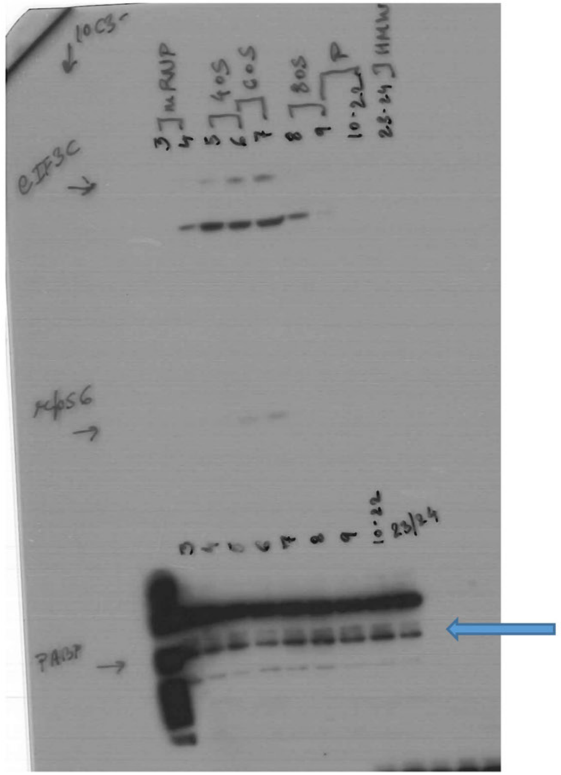

Supplementary Figure 7c

Figure S1_b

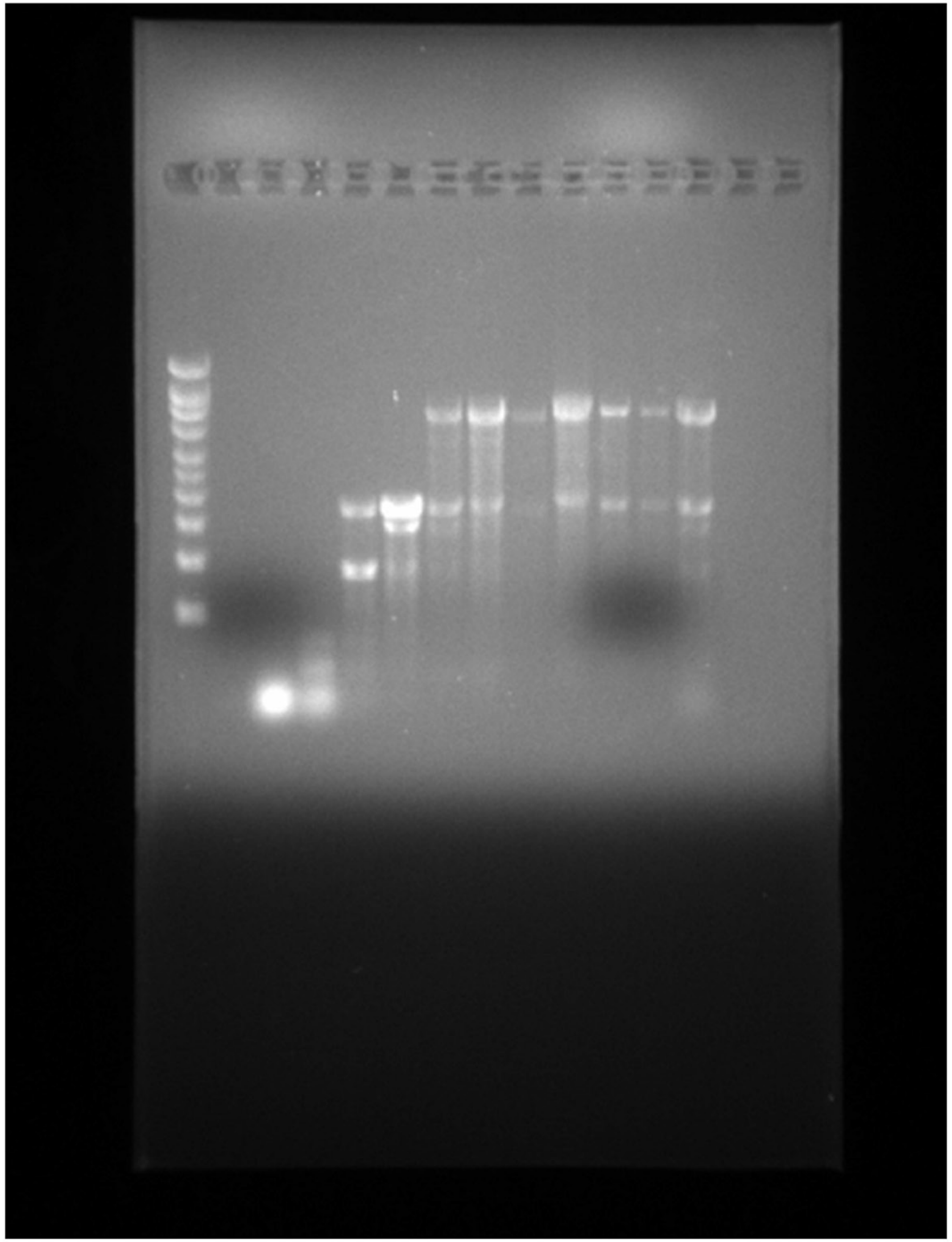

Supplementary Figure 8

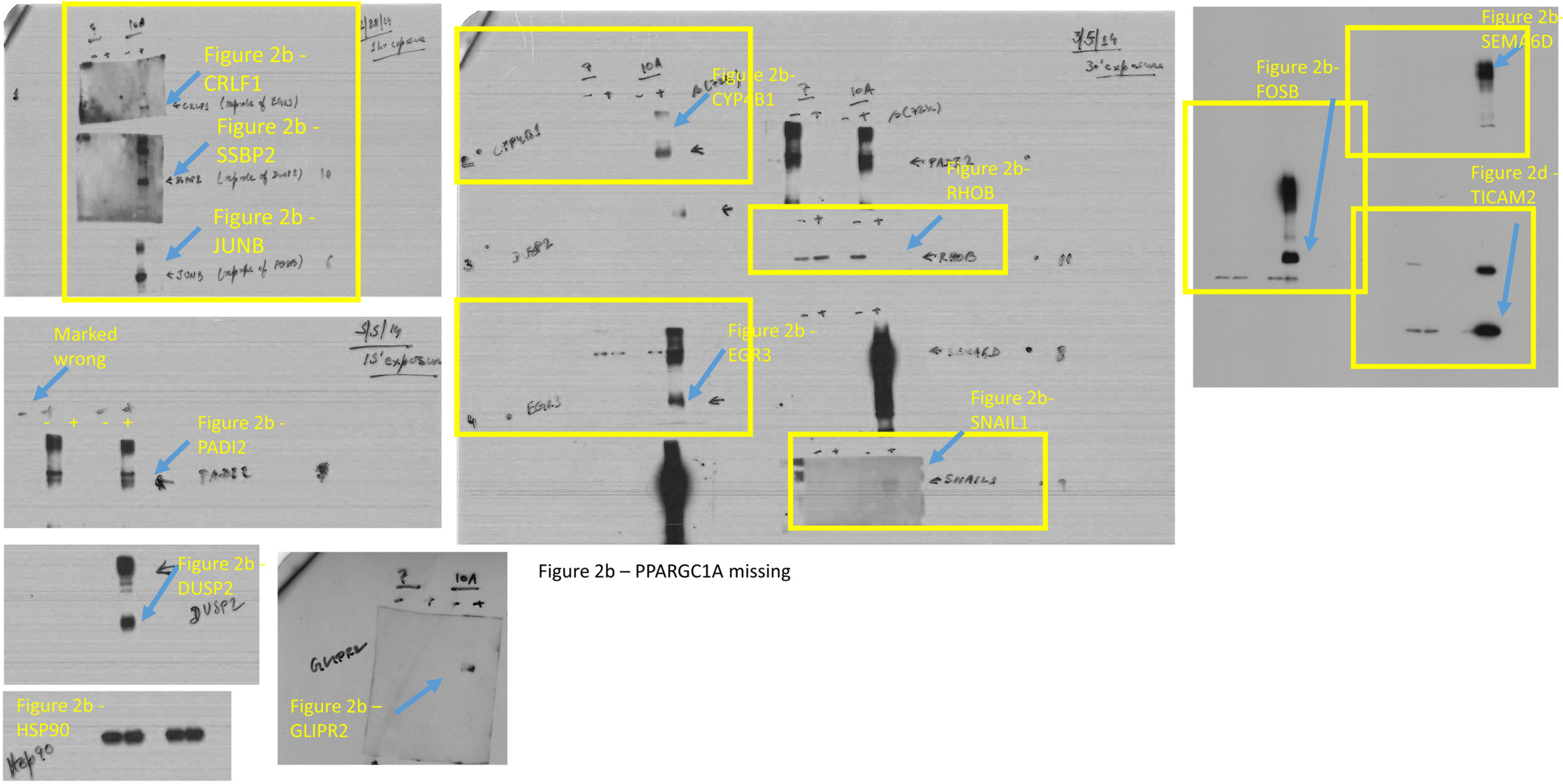

Figure 2b – PPARGC1A missing

Supplementary Figure 9a

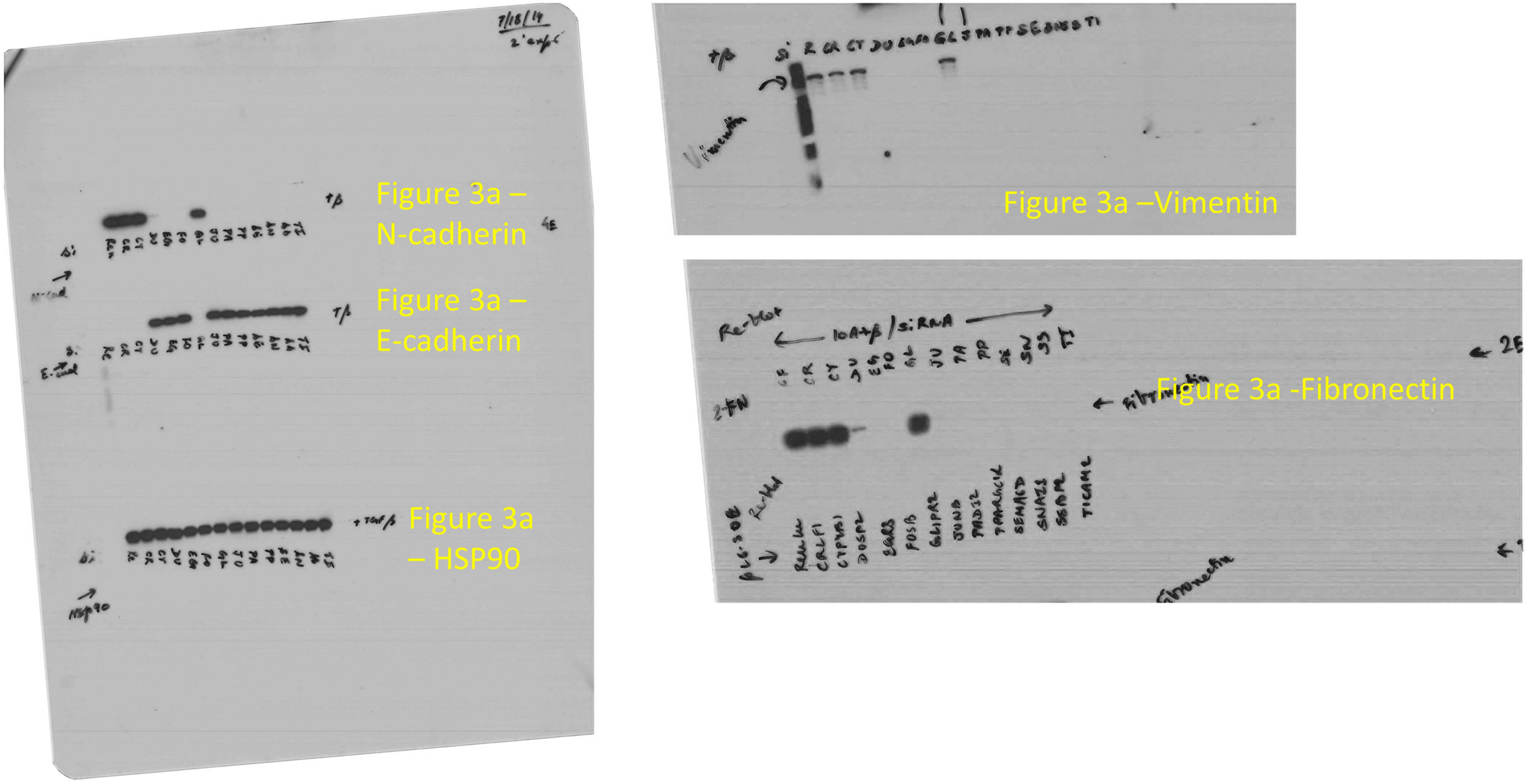

Supplementary Figure 9b

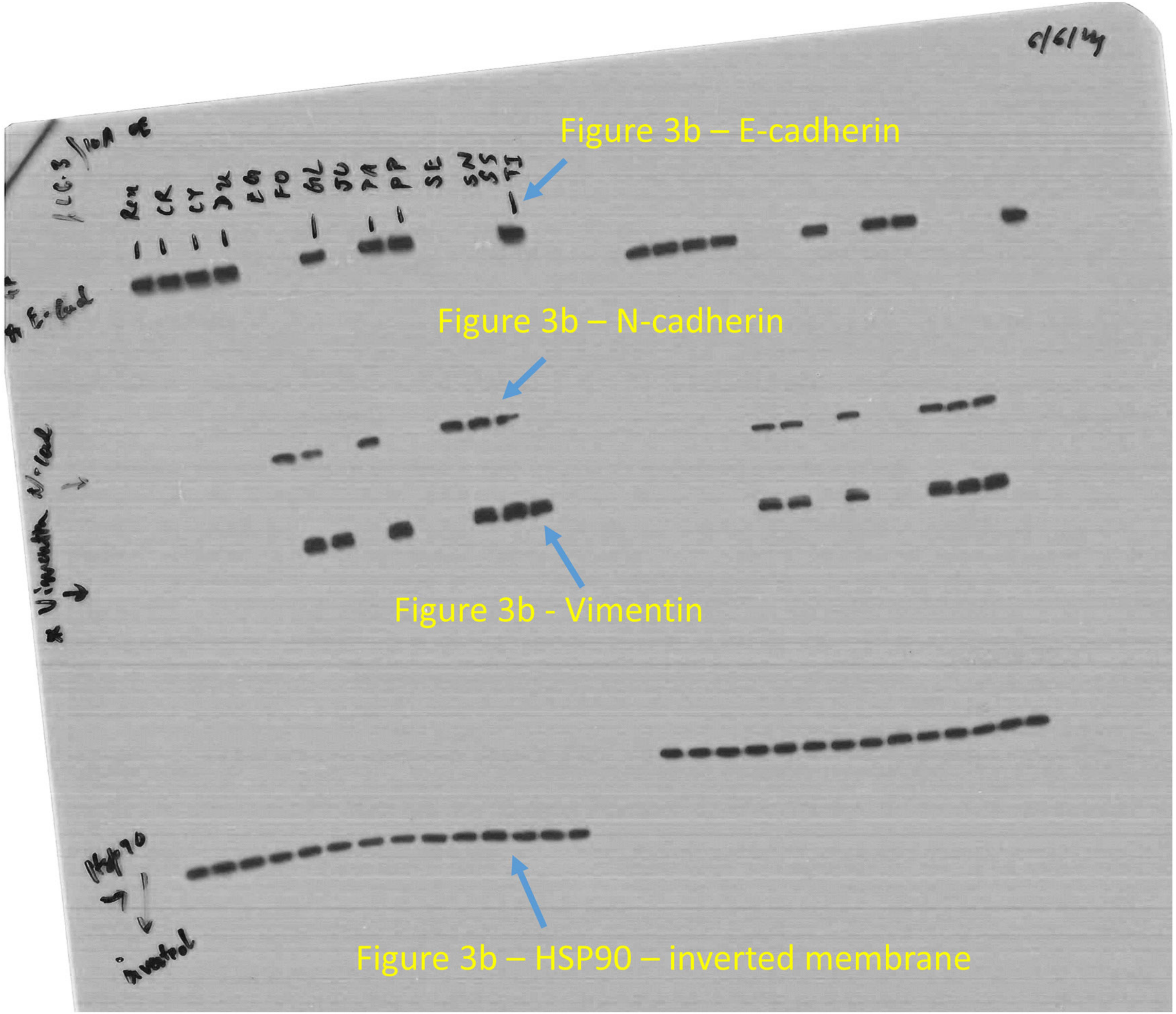

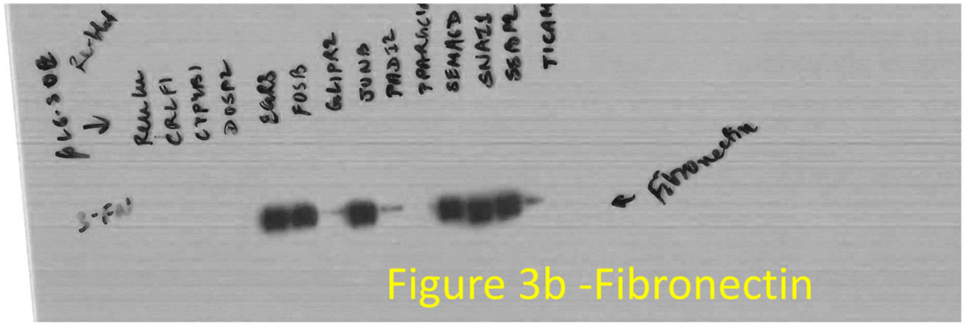

Supplementary Figure 9c

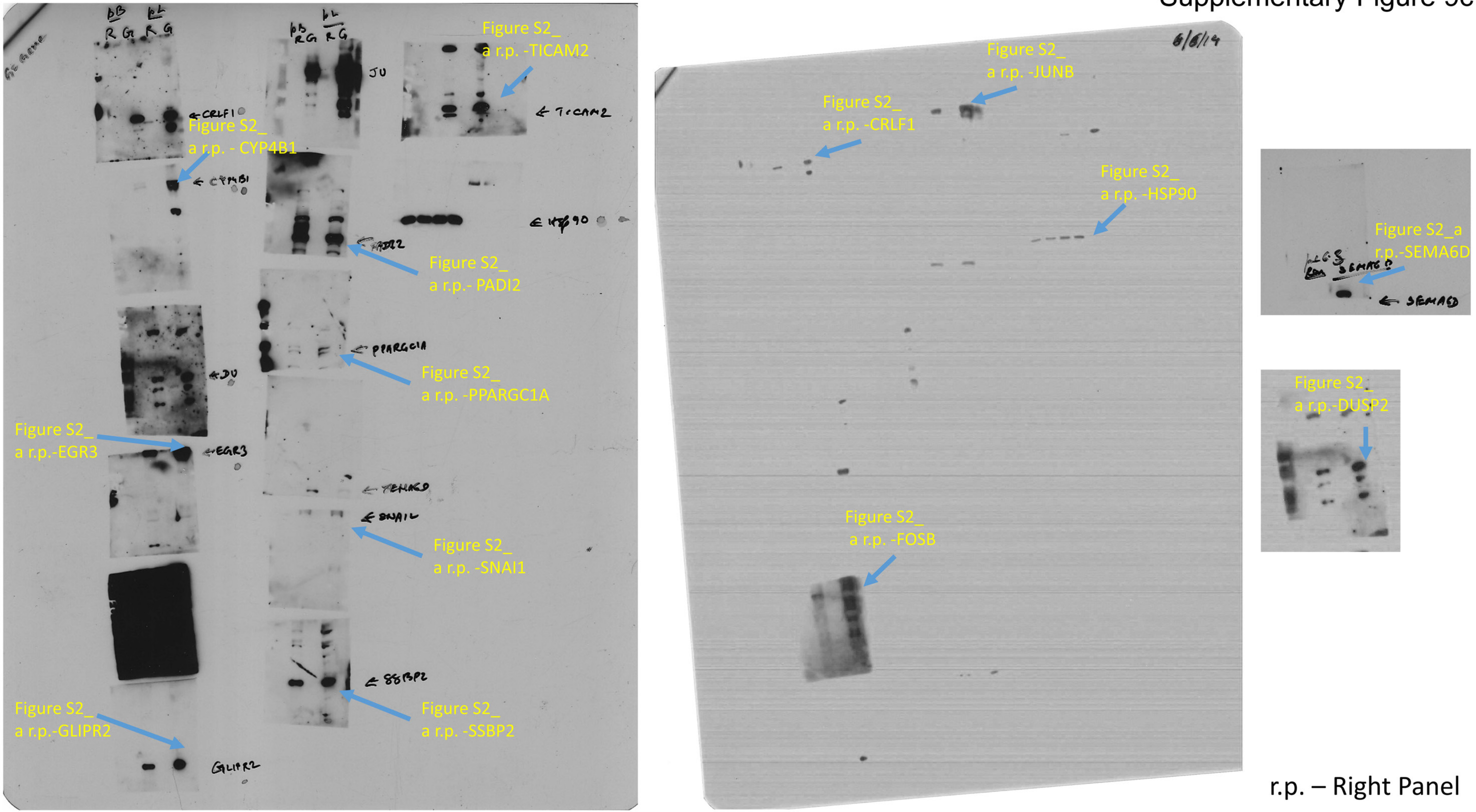

r.p. – Right Panel

Supplementary Figure 9d

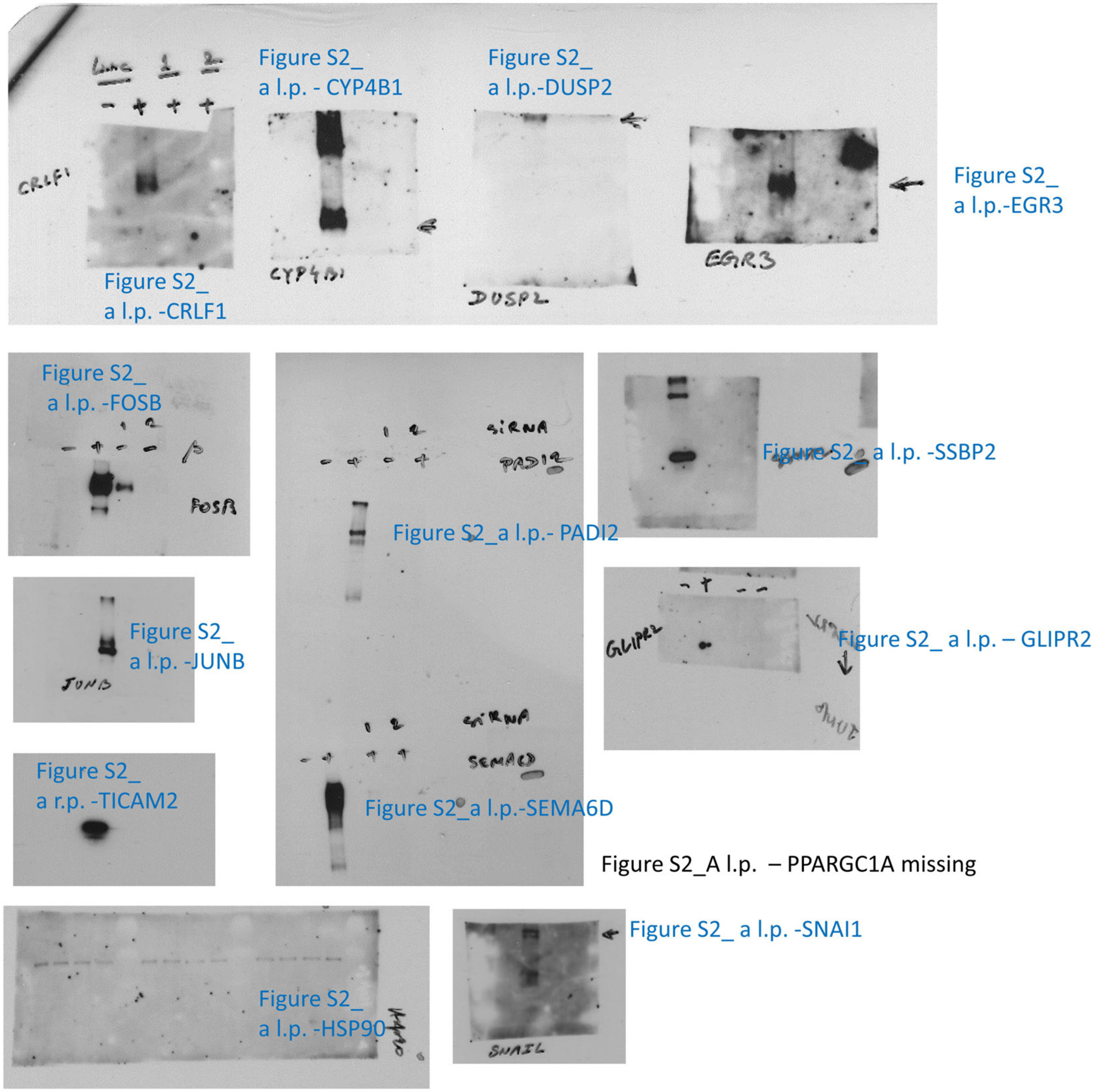

l.p. – Left Panel

Supplementary Figure 10a

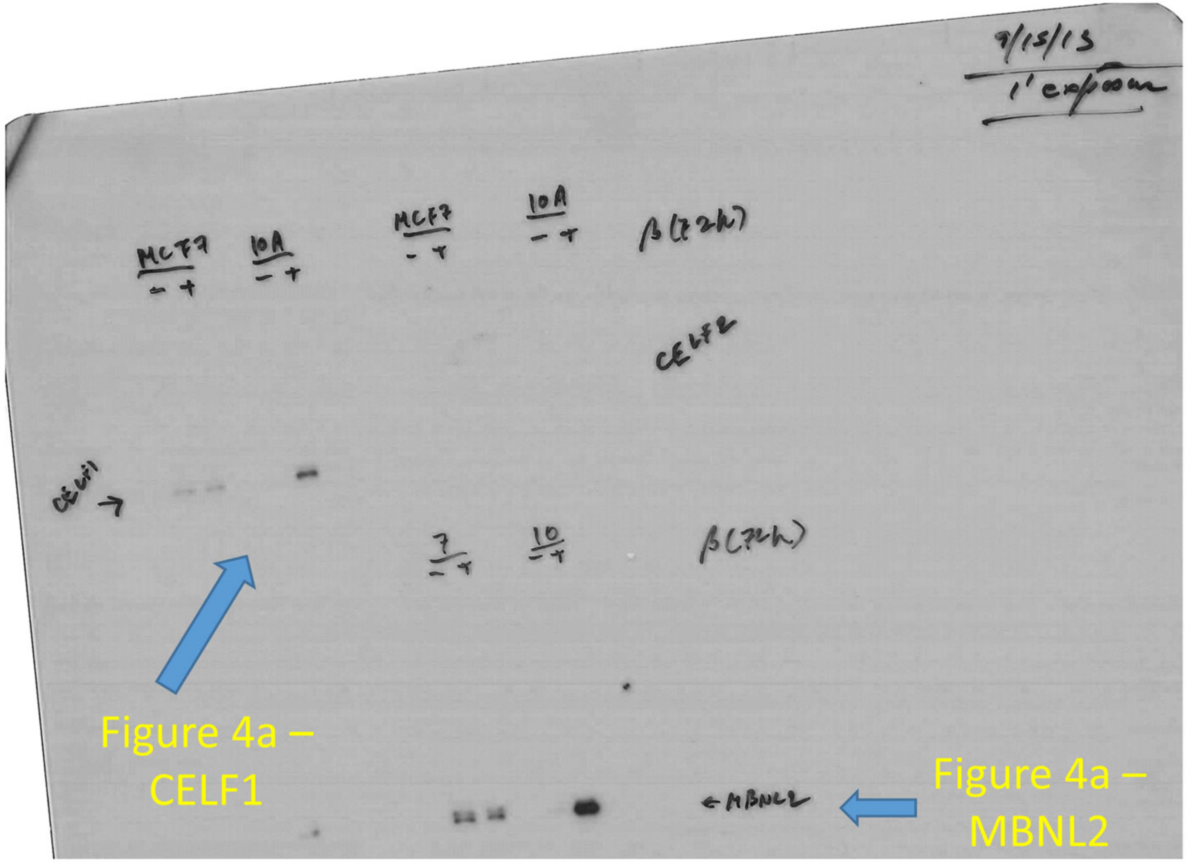

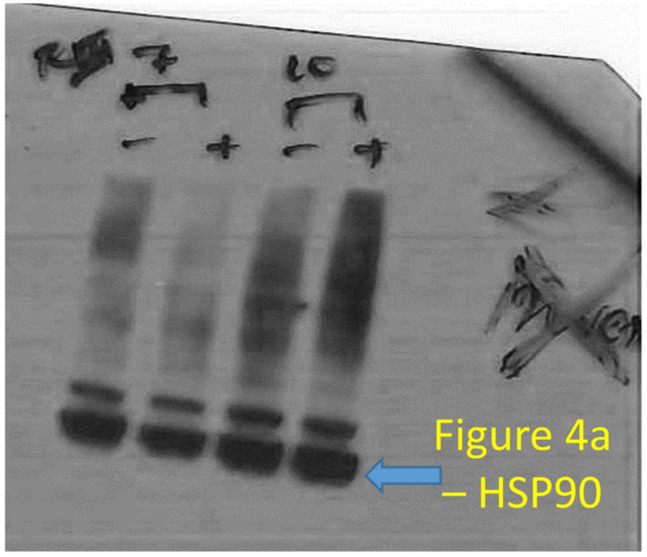

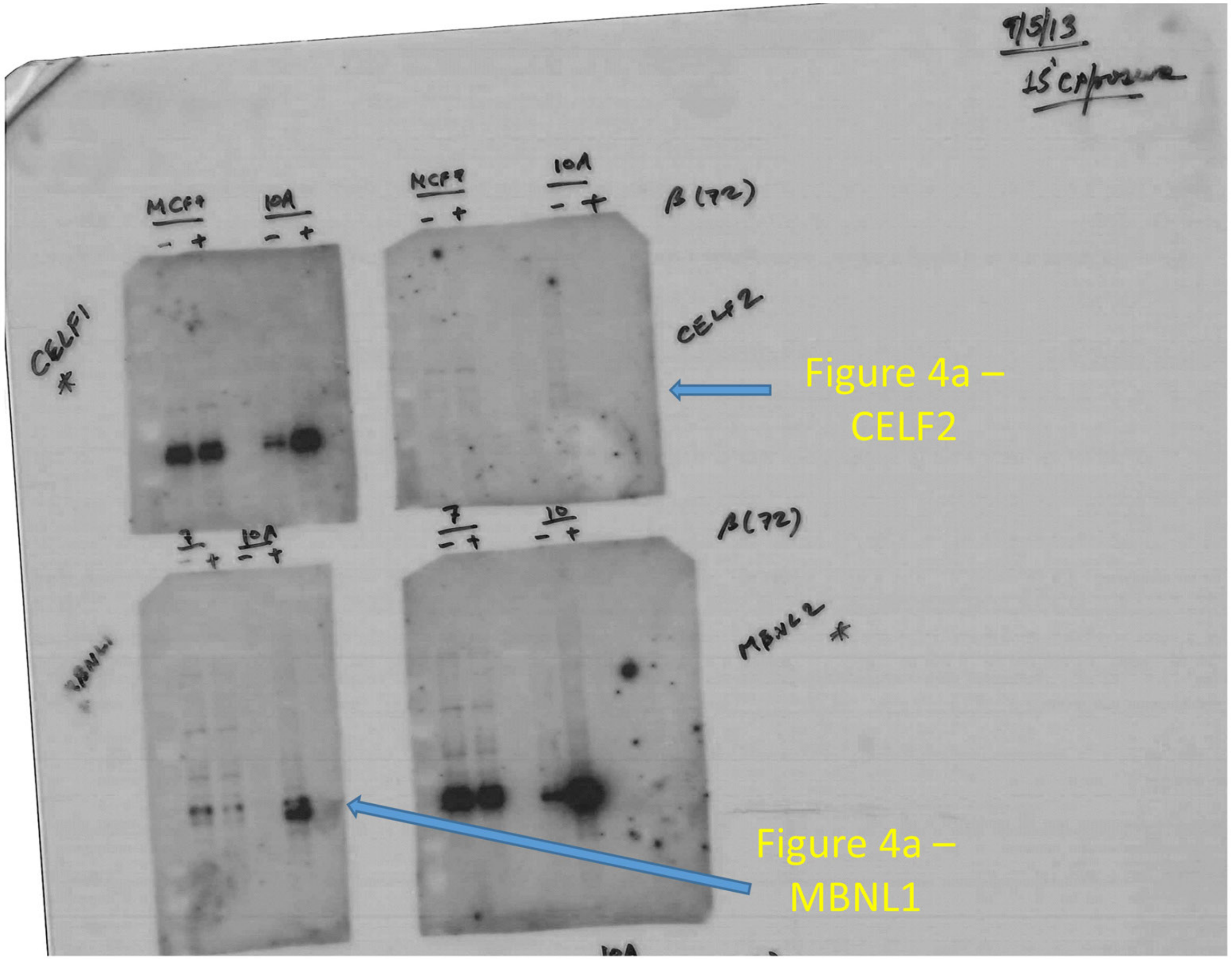

Supplementary Figure 10b

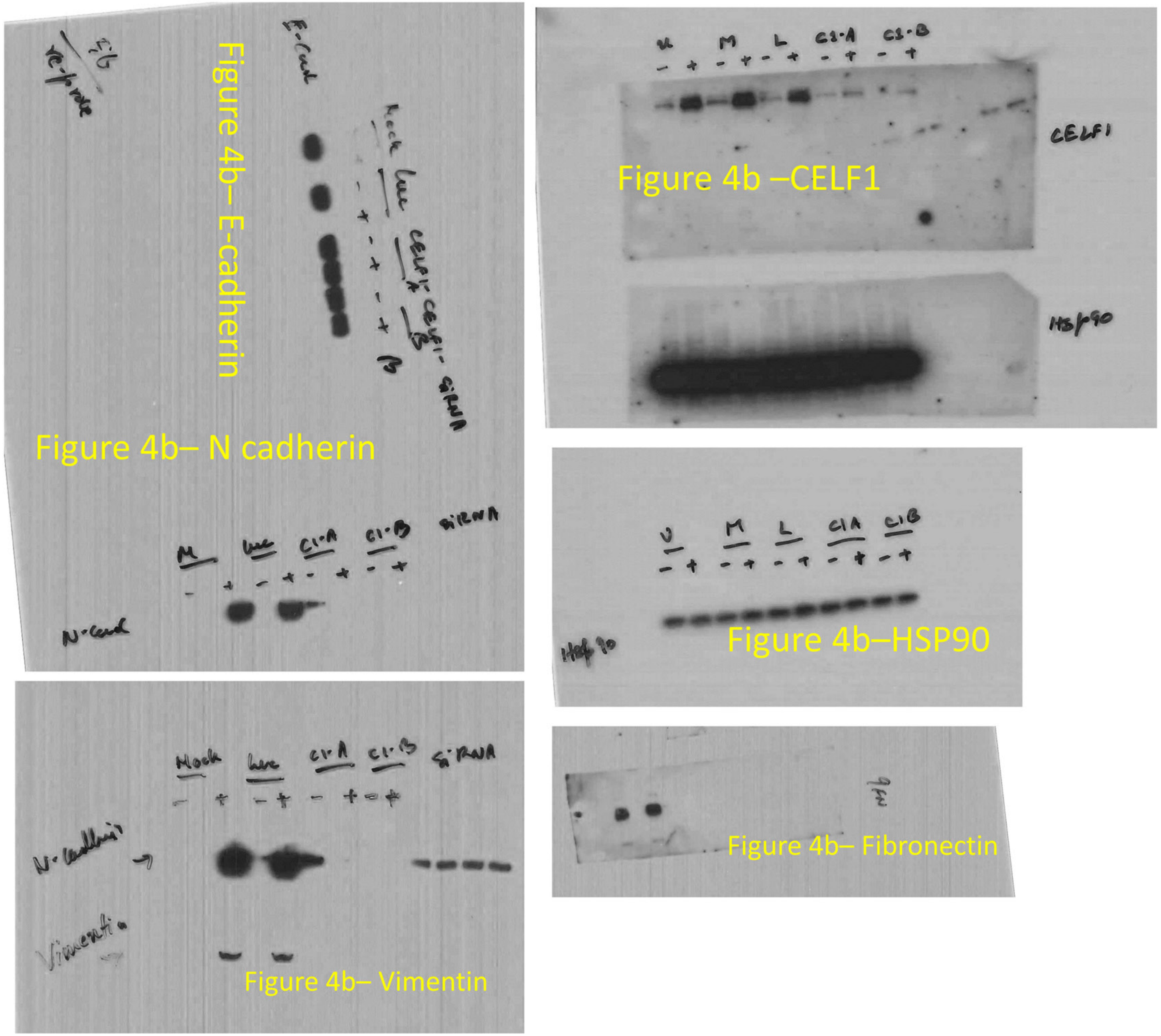

Supplementary Figure 10c

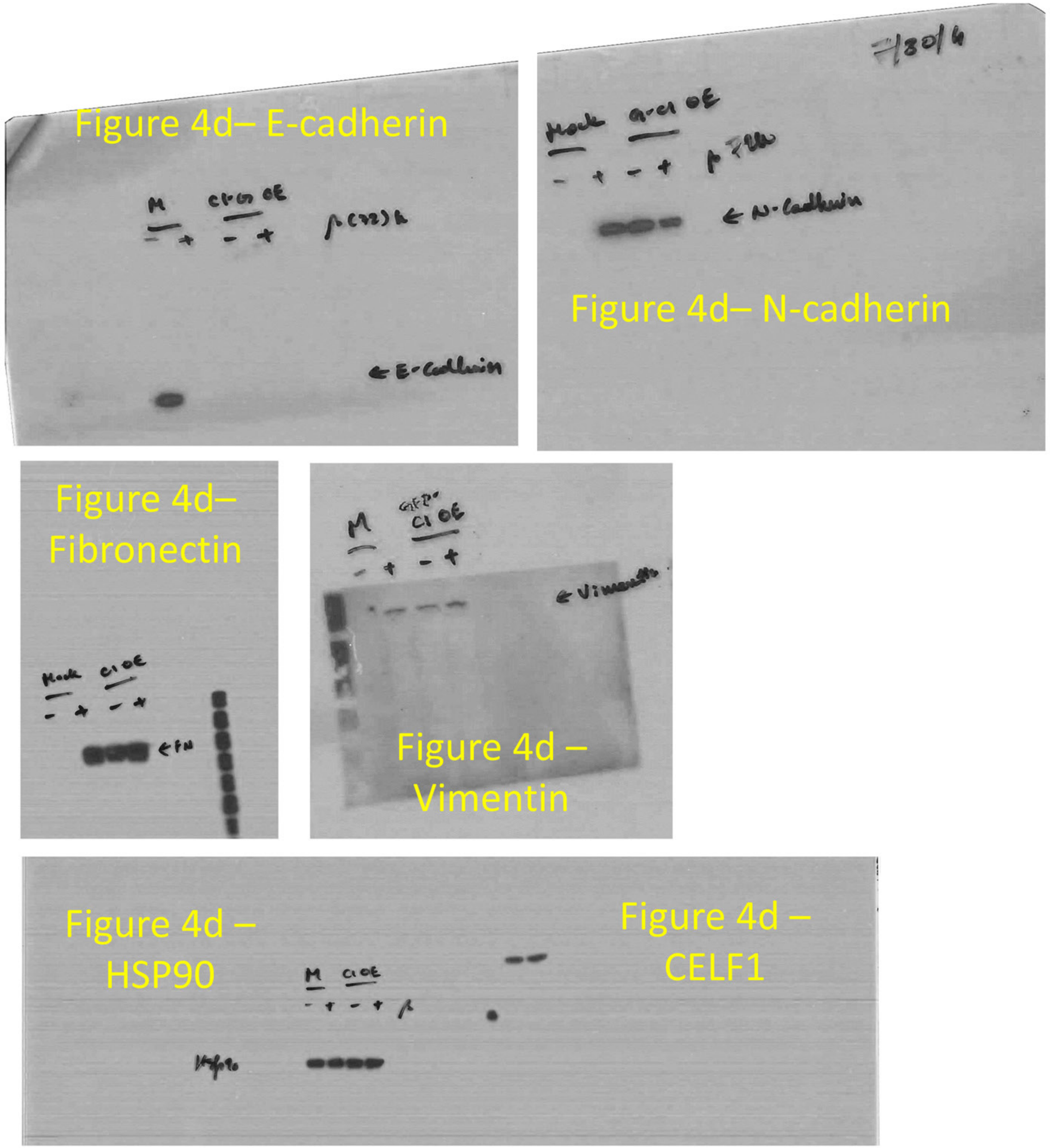

Supplementary Figure10d

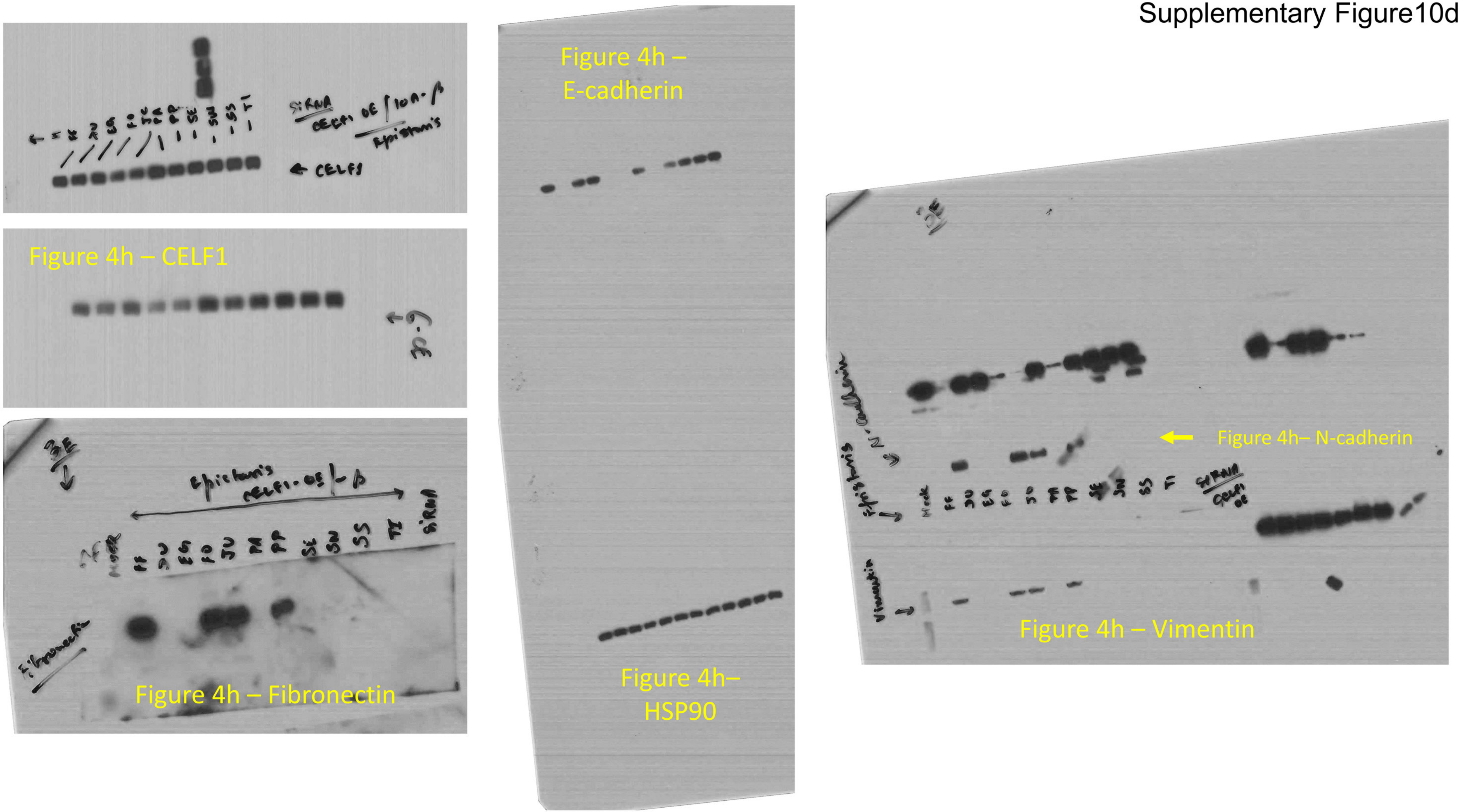

Supplementary Figure 10e

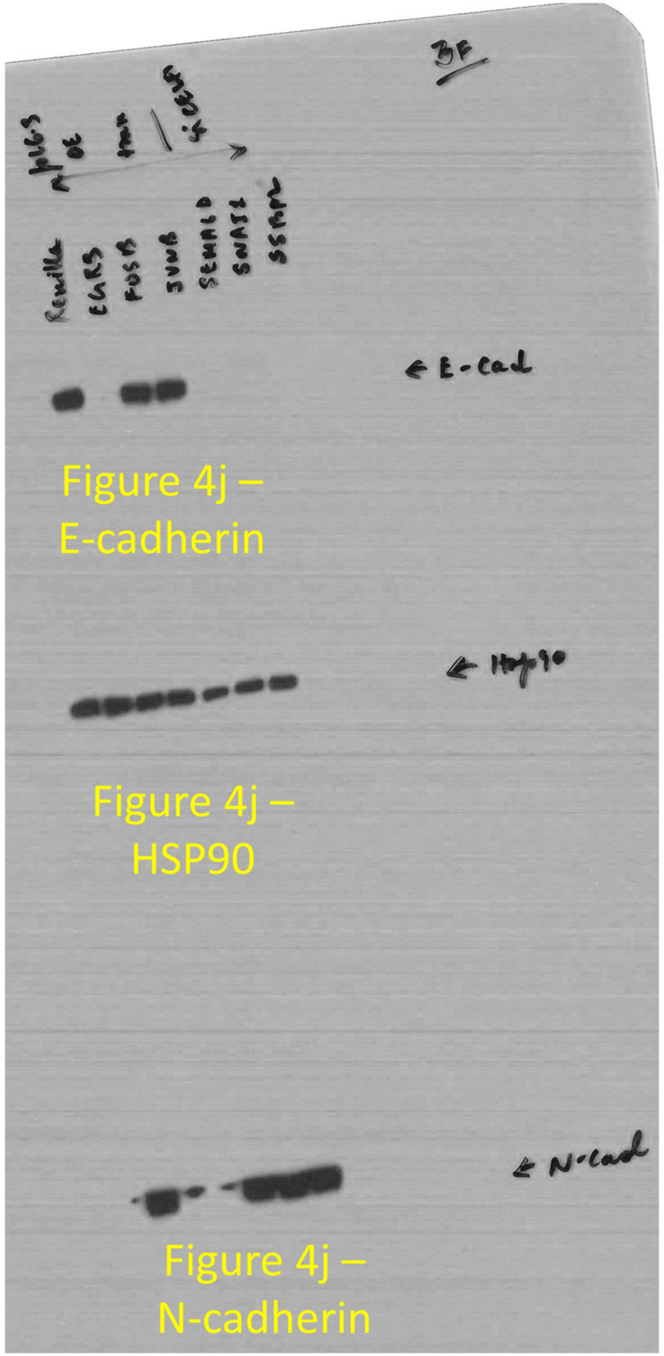

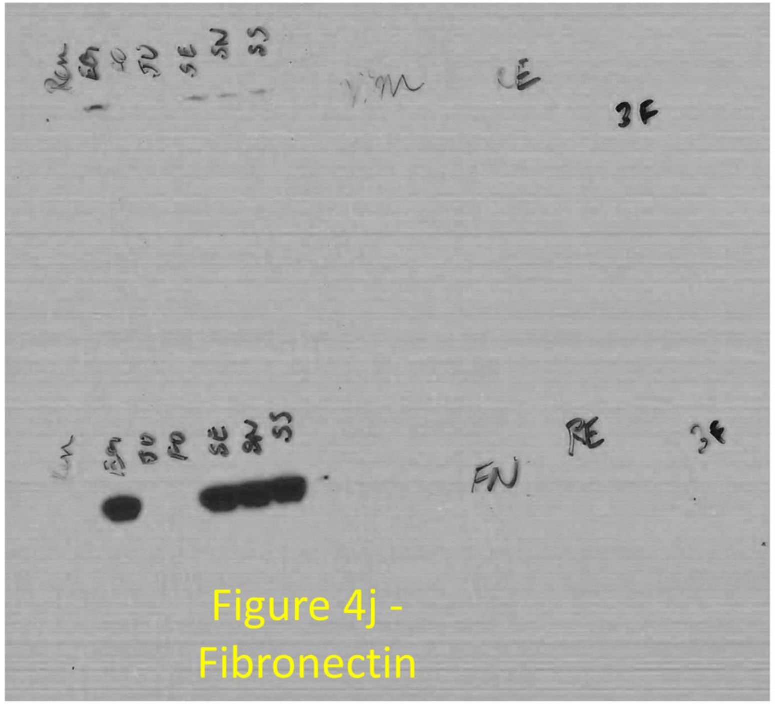

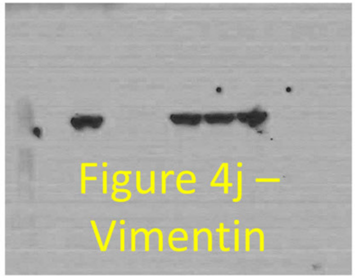

Supplementary Figure 10f

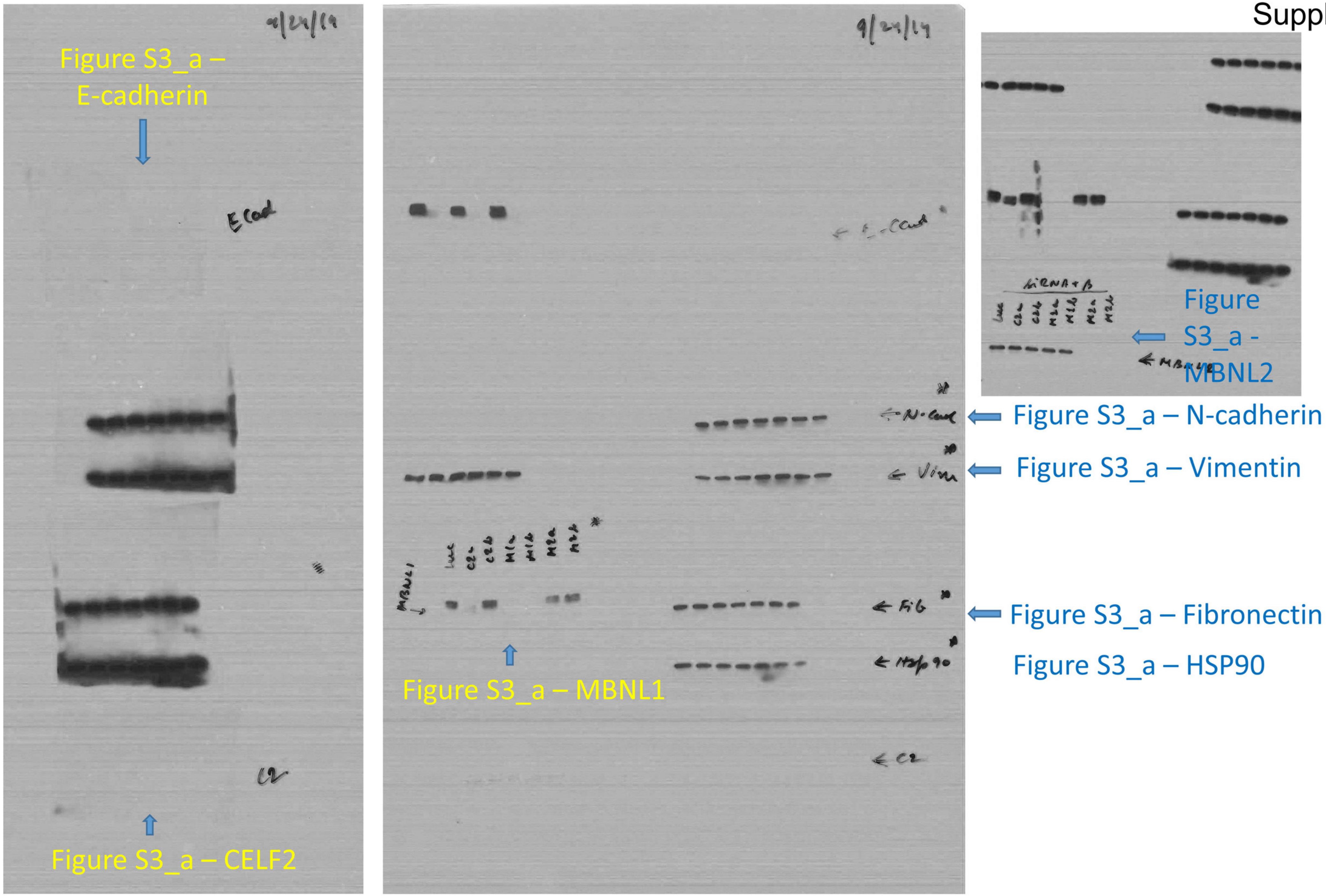

Supplementary Figure 10g

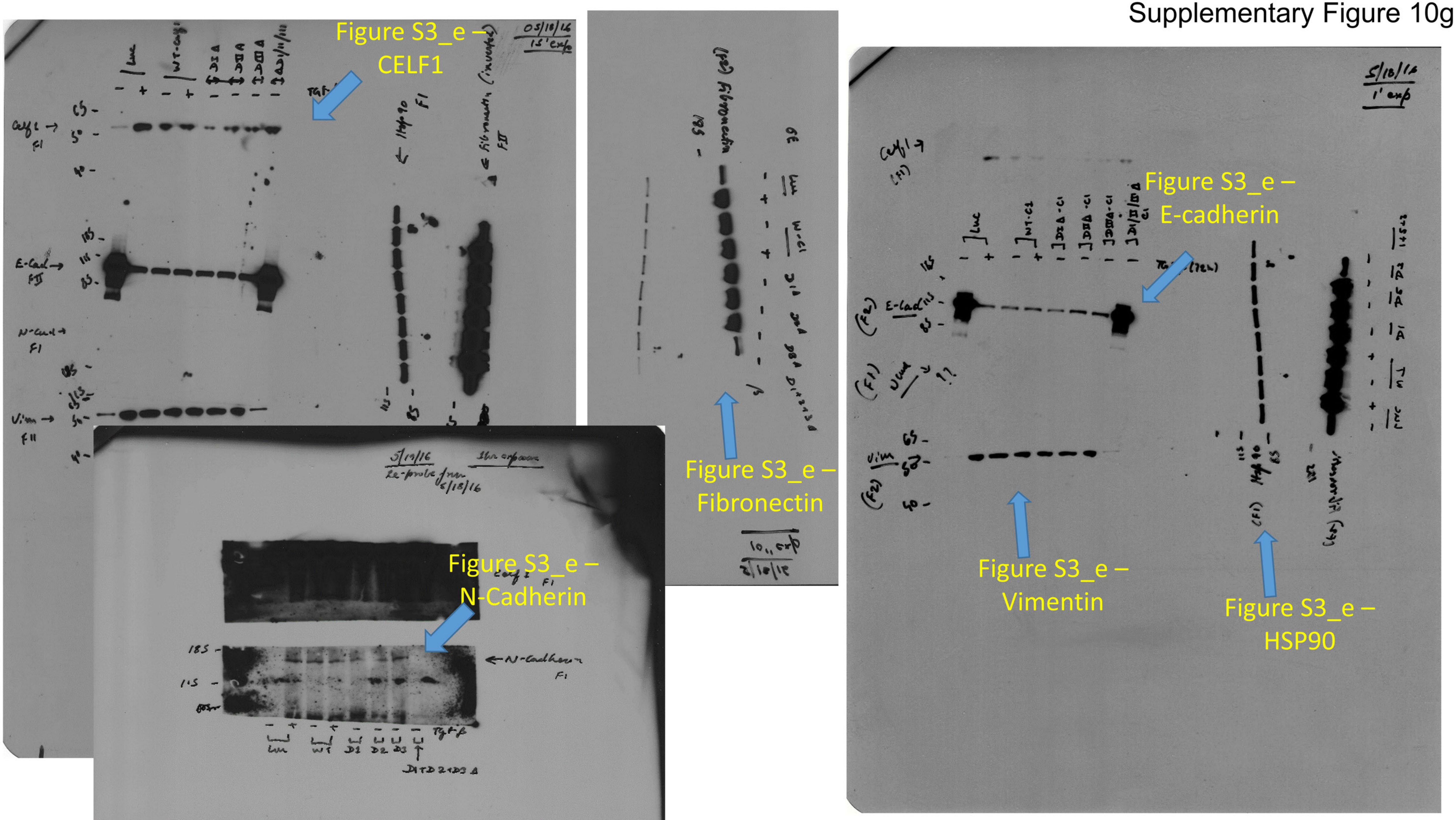

Supplementary Figure 11a

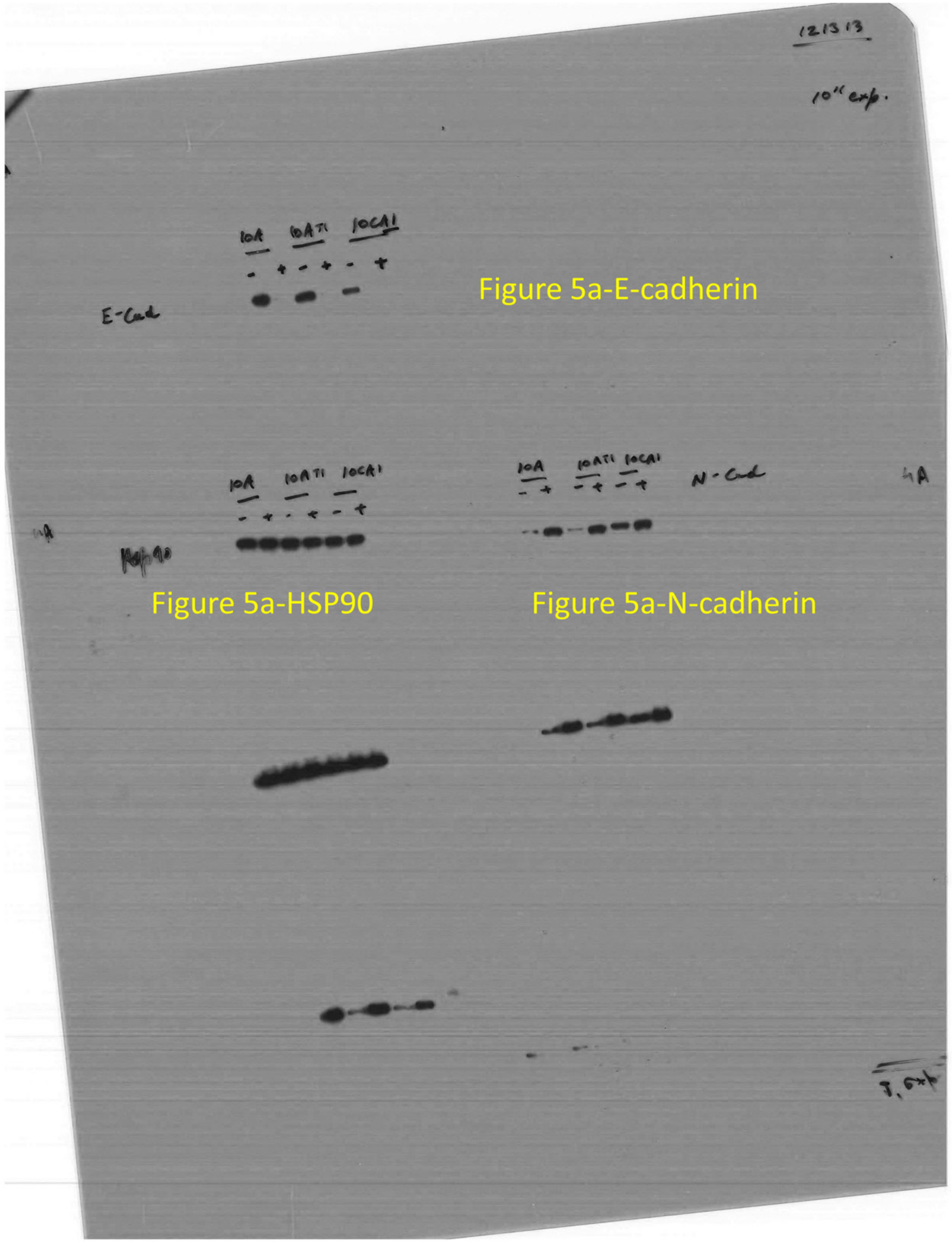

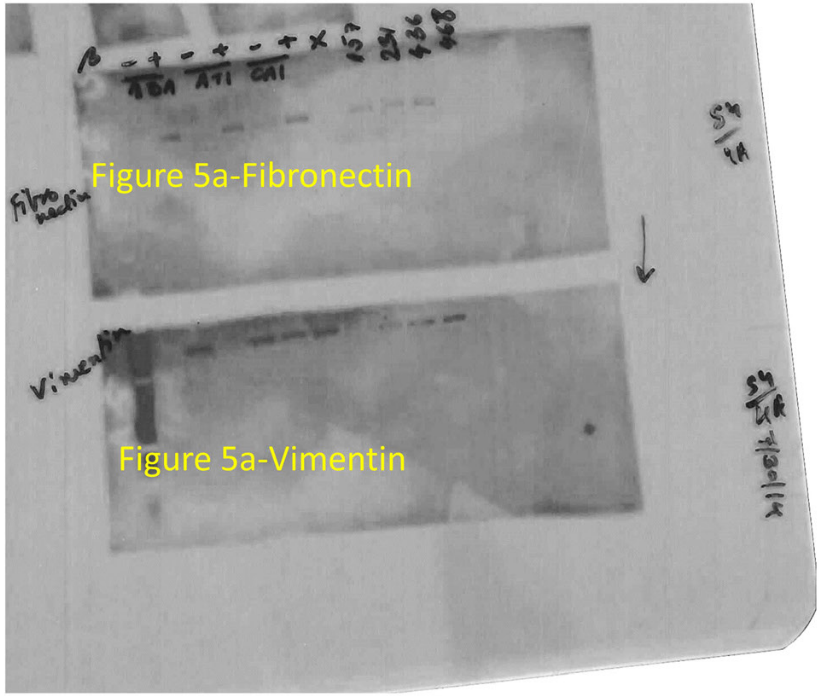

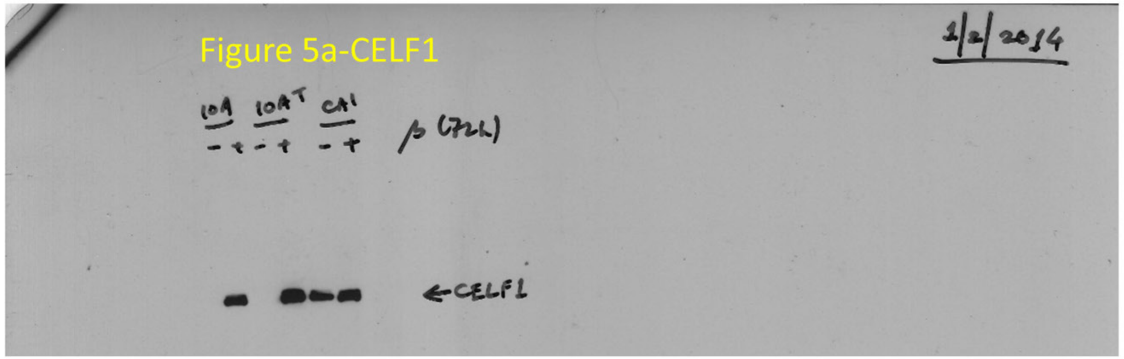

Supplementary Figure 11b

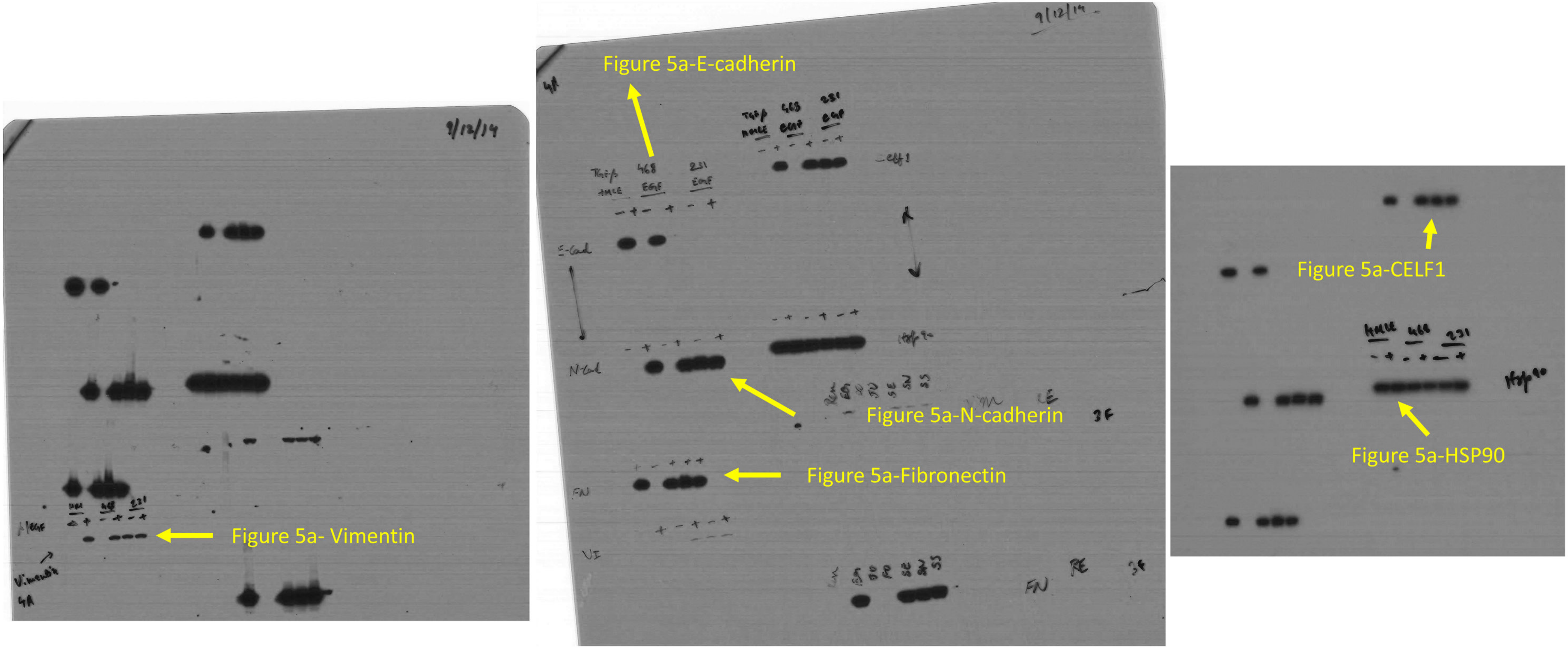

Supplementary Figure 11c

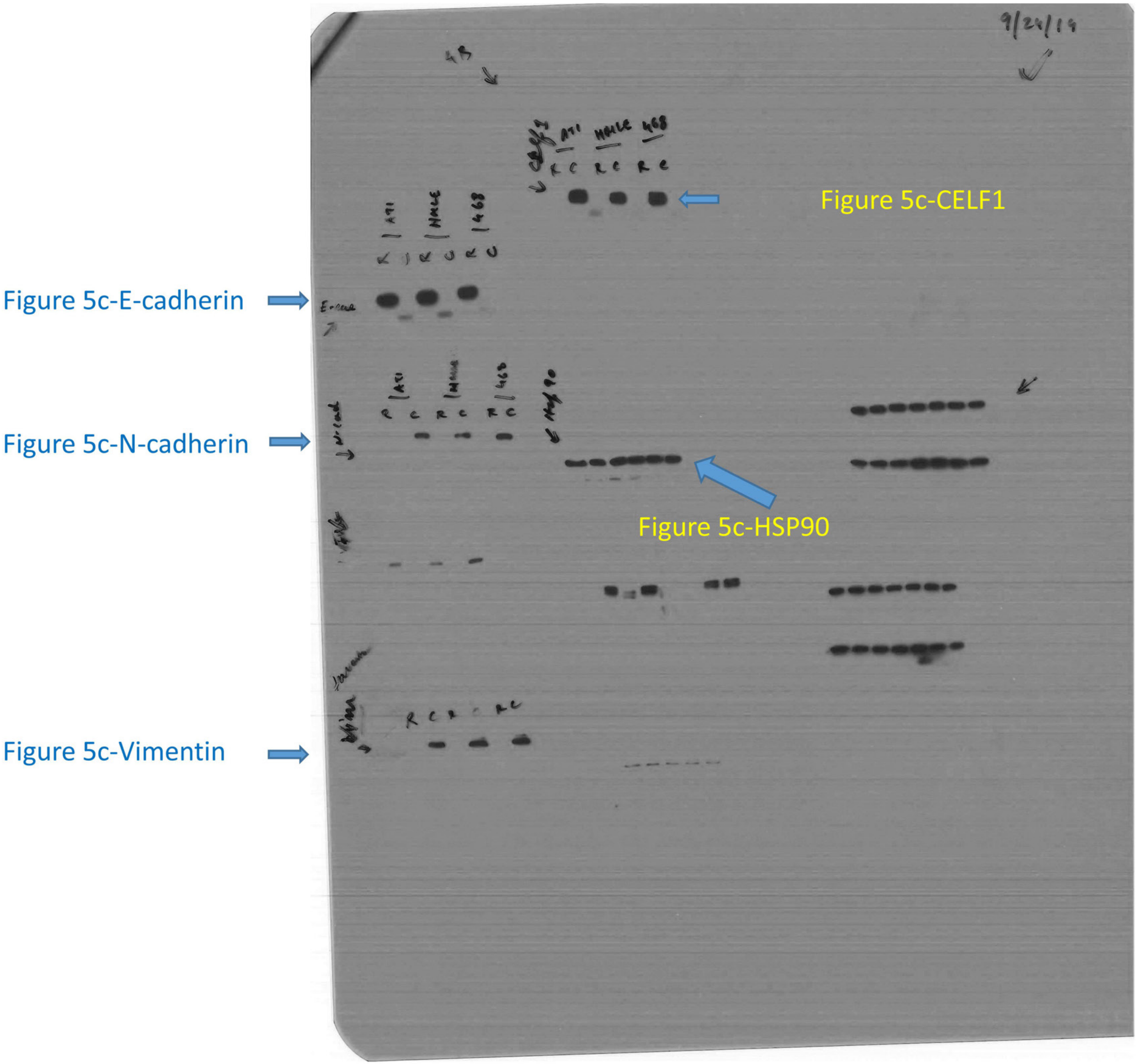

Supplementary Figure 11d

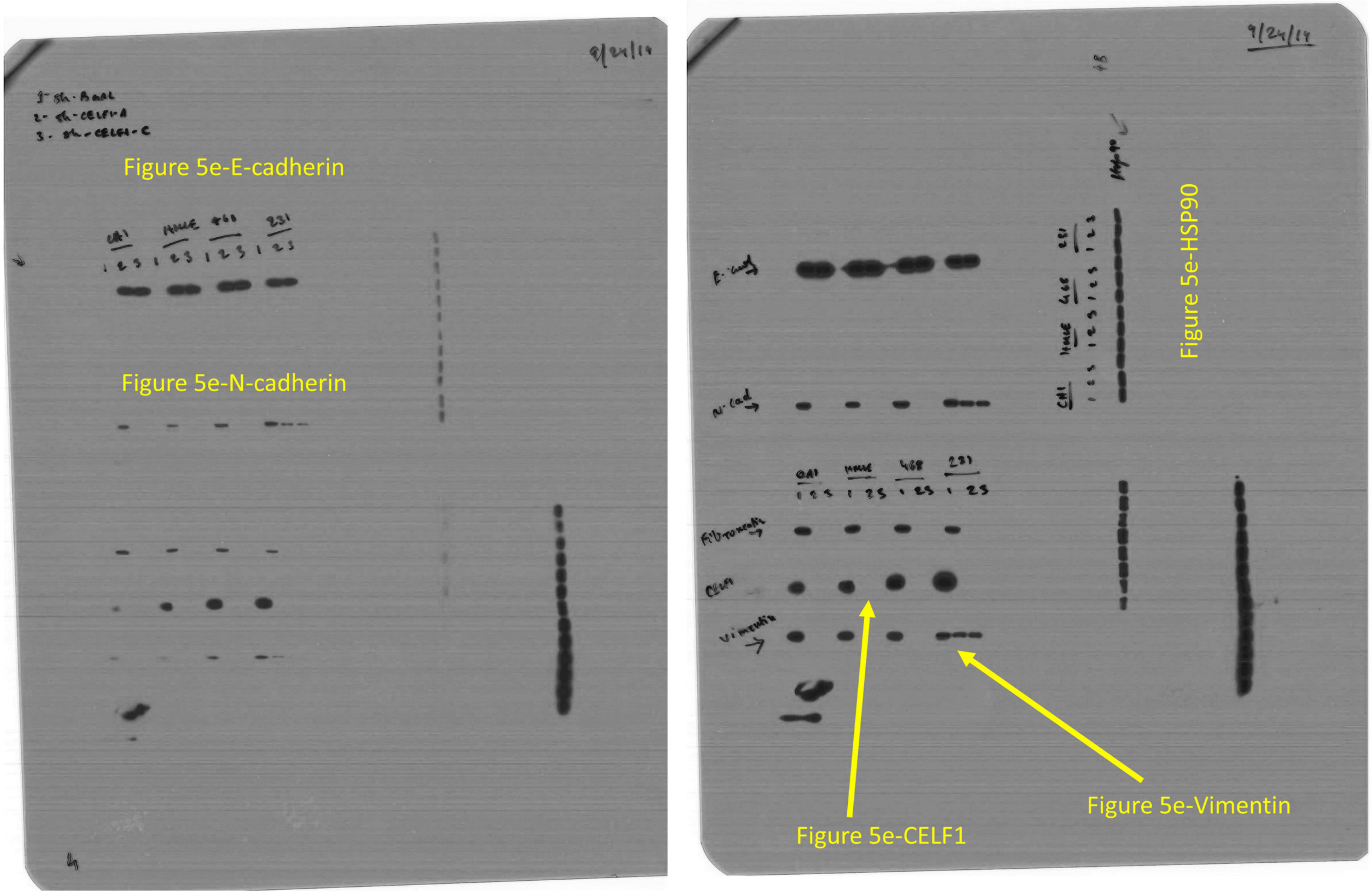

Supplementary Figure 11e

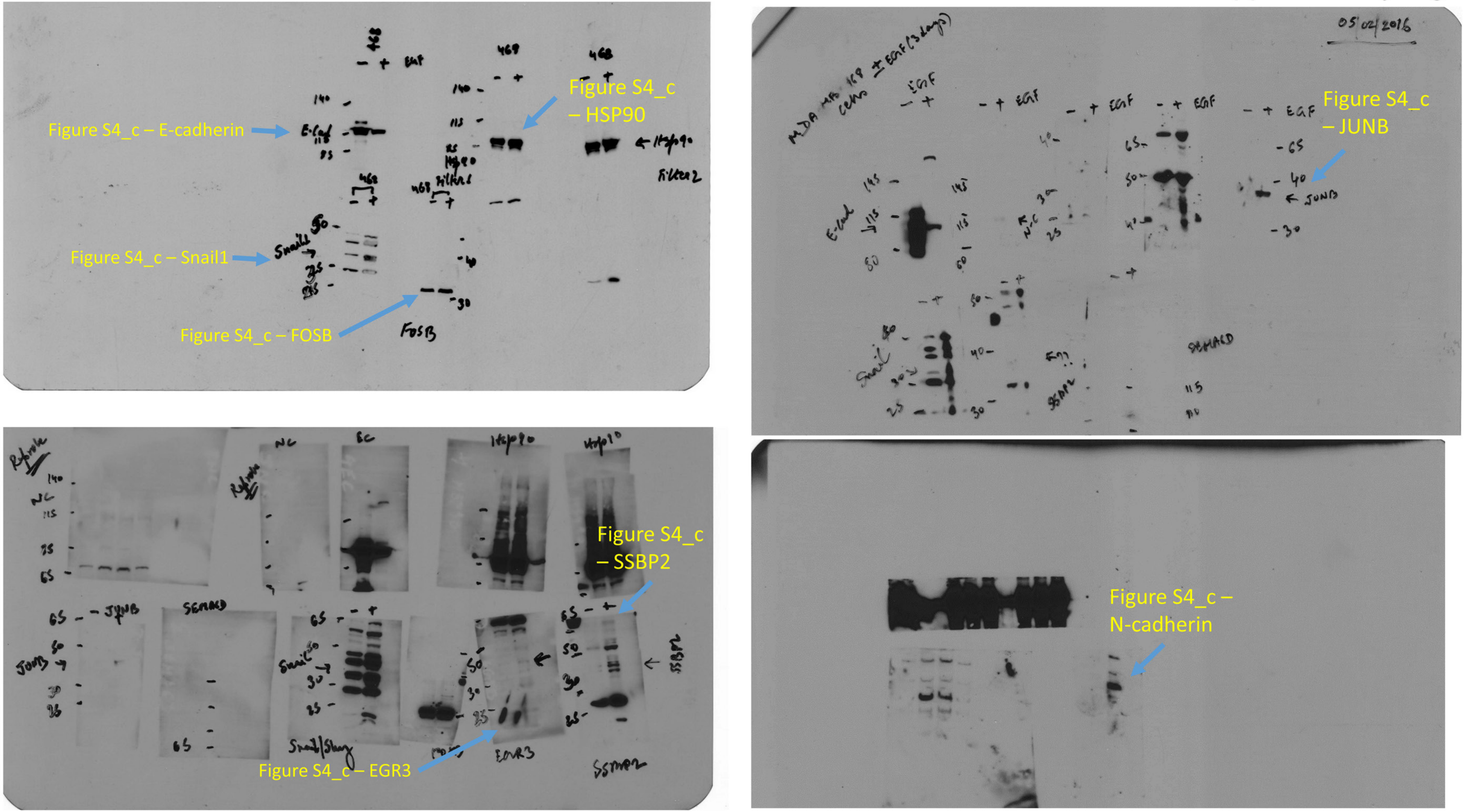

Supplementary Figure 11f

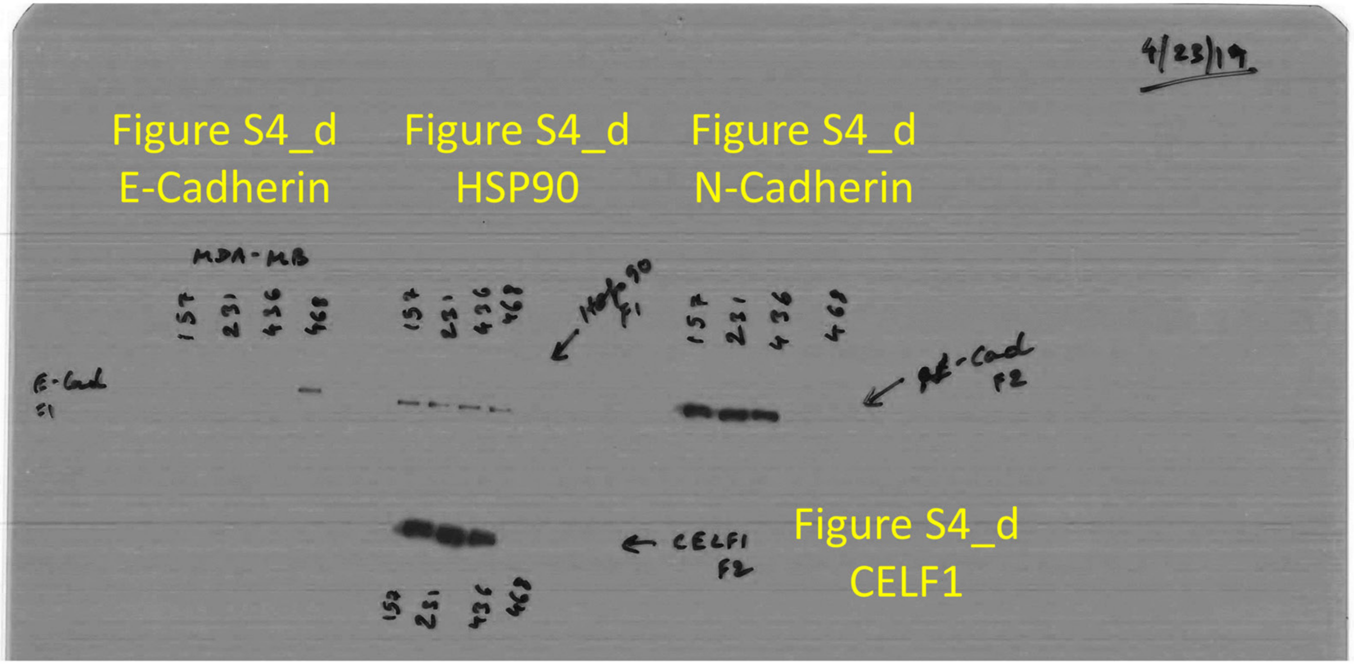

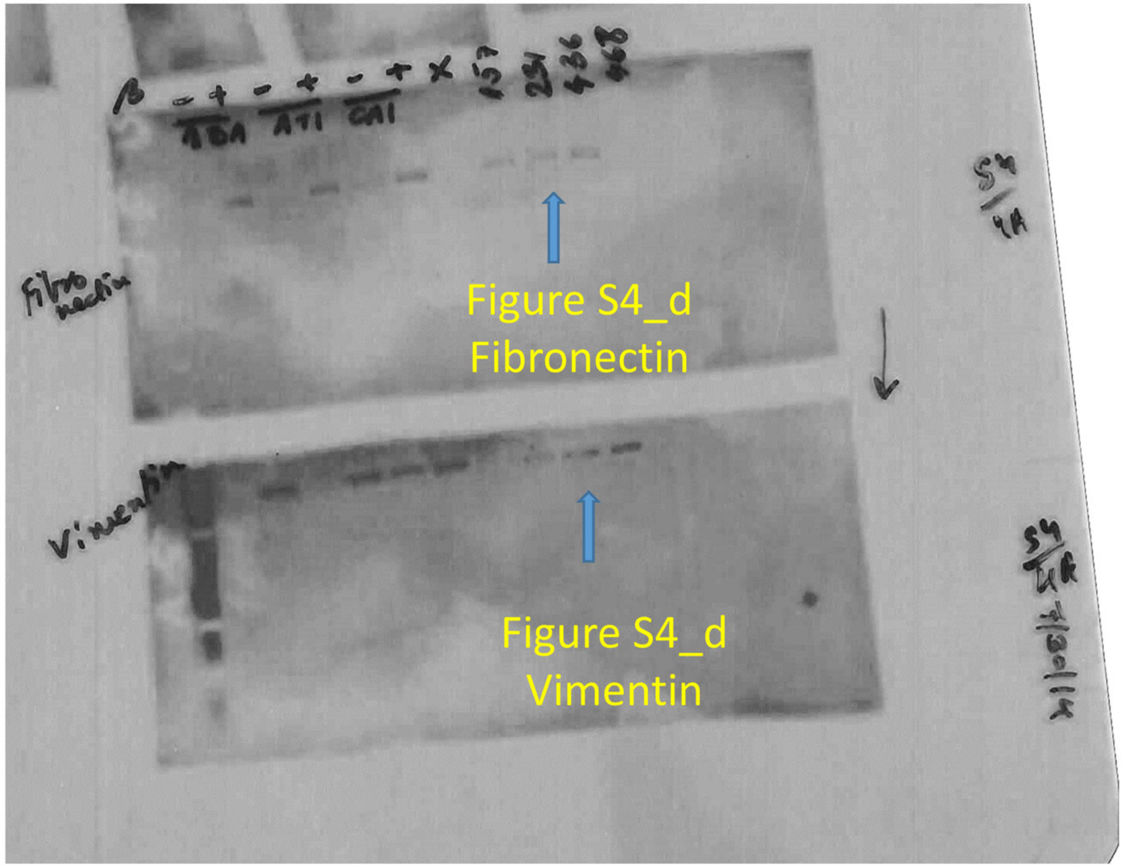

Supplementary Figure 12

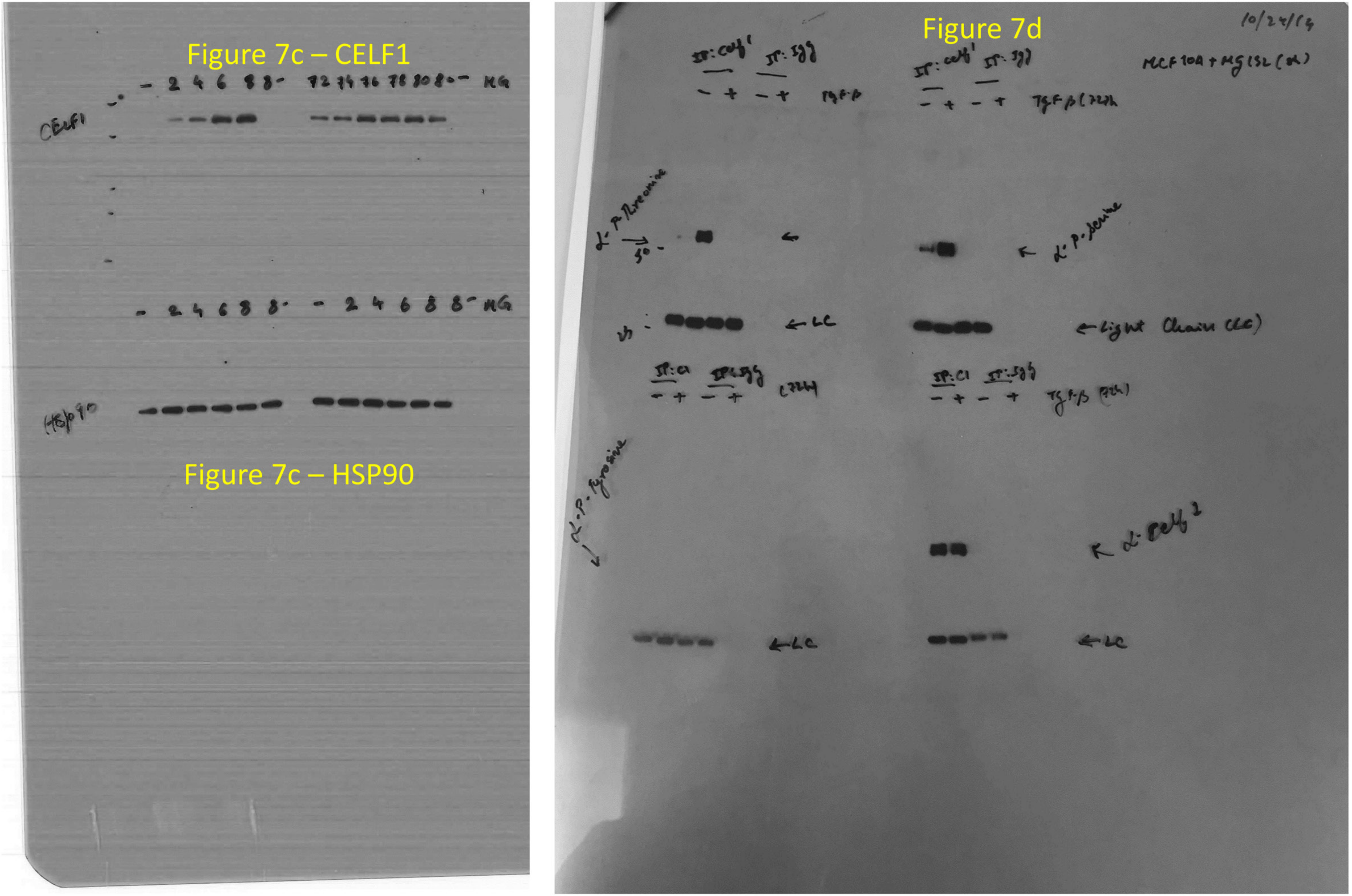

Supplementary Table 1. Oligonucleotides used in the current study.

pBUTRs

**Gene**	**Forward primer (5'-3')** (preceded by *attB2r* -ggggacccagctttcttgtacaaagtggt)	**Reverse primer (5'-3')** (preceded by *attB4* – ggggacaactttgtatagaaaagttgggtg)
ADSSL1	TTTTAGTCACAGACTGAGCTGATC	ACACTGCTTCGGCTGGAC
AGAP6	GTGTAGTATCTGTTTTATTTGACTGCAG	GGCAGAAGGACATAAAATACGTCTTAT
ALOX5AP	CCCTAACTCTCTGCTGAATATG	TTAAGAAAACACAAACCTGGTC
ARHGAP25	GCTTAAGGGTCCCAGGA	TGAATATAATTTTCTCATCAATTTTGCTTGCATA
ASF1A	ATGTGACCACCTACCATCC	TTCATTTTAATATTTTAACATTTTTCTTTTAATTGCAATTAGACAAATG
AZGP1	AGCTAGGAAGCAAGGGTTG	AGCTTCTACAGATTAGACAATGGG
C12orf35	AAATAATTACAAGATGTGGTTTTGTAATTGCC	TCAAGTATGAAAAGTAATGTTTAAACGTTGTC
C14orf142	TCTTAACAATAGCCTTCATGACATTTAAAAGAT	TTTAAAAGGATTCAATAATCTTTTTAAAGCAAAATCAAAGTAT
C16orf87	CTCTGATACTTGTTTGCAAAATCTGC	TTTTCTTCCAGATTGAAAGTAACAATTTAAATAATTATCAG
C4A	GTGTGAGGGCTGCCCT	ATGCCCTGGTCCCAGG
CBWD6	ACATAACACTAGAGGCATTTCTTATCAAAAGG	TCATGTCATAAAATGGTGACACTTACTT
CCDC59	TGTTAAACATTTTGTTCCTACAGGTTAAAATATCTG	ATTCAATATTTGGCAAGTATTATTGGTCAGC
CCNL1	CGCTGACTTTCTCTTCCTTTGA	TCCTTGTAAAAATCTTTACACATGCAGAC
CEBPZ	AAATGAGTTATTAATGTAAATTATAGATTAAAATTCTACTTACATCTAATT	ACAAATCCACTGAGAAGTCTGG
CRABP2	GAGTGAGTGGCCACA	AAAATTAACAAATAAATATTCTAAACTGTA
CRLF1	AGATAAGCTGTAGGGGCTCA	TAAAAGGACTCTTTTGGAGGG
CXCL14	GAATAGGGTGAAAAACCTCA	ACTTTCCATCTTAGAAAGAATATG
CYP4B1	AAGTAGCTCTGATGAGAATG	ATTTTGTACAGTCTTCATTTAGG
DBNDD2	AGCTAGCAGTGGGCCC	CCGTGCTCTGTTCCAAAAA
DUSP2	CACTGAGGTGGTGCC	GACATATCCTGAATGTTCTGTAT
DYNLT3	CTTTAACTGACTAAAAATGTTGGGCTAAAGC	ATATTTTAACTAGAAACAGAGCAGATAGCAAG
EGR2	CCTTGAGATGAGACTCAGGCT	TACACTATAGTCACAAACCATCCA
EGR3	GCCTGAGGATCGGG	AAAACAAAGGAAAACTCAATTATC

EGR4	CTCTGAGCAAGAGATGGGTTT	GCAAAAATAAACAGTTTTGTCAACTG
FAM206A	TCATGAGGATTGACATGGAACAAAAA	TAAAAACAGTGATATTGACATGTATGTTATAGCC
FGFR1OP2	AGCTGAAGAGTTTCTGAGTCTGT	AGCAGGGATAAGAAGTATCTGAATGAAT
FOSB	CTGTGAACTCTTTAGACACACAAA	GGCAACAGTGCAGAACCAA
GGCT	CTTTAGAACATAACAGAATATATCTAAGGGTATTCTATG	CCTCTAAAAACAGTATACCATCTTTCCAAT
GLIPR2	AAGTAACTTGTTAAATGTAATGGG	GTCTTCTTAGTCAATATCCCT
GPR1	CAATAAGTTATTACTTTTCCACAA	TATCTATTTTATTTATTTATTTTTGAGACA
HDGFRP3	ACCTAACTACCATAATGAATGCTGC	GTCAGCTTGCTGTGAACAATTTTT
HIST1H4K	GGTTGAGCGTCCCTTTC	TGGGGGCCCTAAAAAGG
HSPB1	AAGTAAAGCCTTAGCCCG	TGAGAAAAACAGAAGATAAATGTATC
ING2	AGGTAGTAAAGGCCATCCACATTT	ACACCATTAATACTAATGATTAAAAATTATTGCATAGTCTTAT
INSIG2	GAATGAAGAAGGCAAAAAATATCTTTTGTACA	ACGTGTTGCTTTTAACAGCAATTTT
JUNB	TTCTGAACGTCCCCTG	TAAATAGATTCAATAAAAAGAACAAACAC
KLK7	CGCTAACGCCACACTGA	TTTCATGTTATAAAAGTGCACATTCAAGG
KRT4	CGATAGAGGAGACGAGGT	GAAGATTCACCTGCAGATGG
LTF	AAGTAAAACCGAAGAAGATGGC	TCTTGCTAAGACGACAGCAG
MME	TGGTGATCTTCAAAAGAAGCA	TTGTATTTTTGAGACTGGAAACTG
MRPL42	AGATGATGCGGAGGTTCC	ACTTTTGGAGGCCAAGGTG
MTRNR2L5	GTATAAATAAATAGGAGAAGACCCTG	TTTGTGAAGTGGGCCC
NUF2	ACCTGATTAACAAAATTACATGTCTTTTTGT	TAATTATAAATTAATAAAAGCCTACATTAAATTCATCTTATTAAC TACT
PADI2	CCCTGACCTGCCAG	TCATTGTTCTTTAGTCGAGC
PCNP	AATTAAATGATGTTTTGAAATTGGGGTGTG	GGTACTTTAGTAAAGACATTCATCTCAGT
PDZK1IP1	ATGTAACCTTCTCTGTGGCTC	TCACAGAAATTAGGGCCATTTC
PLK4	CATTGATTAAAACTCCTTTCAGACATATAAG	TTTTTATAAAATGCATTTGCAAAAATGTTTCATCA
PNN	CGTTAATGGAAGAAGCCAGGCTTT	AATTACTCCAATAAAAGGATTTTTAAAAAGAGATCT
PPARGC1A	AGGTAACATGTTCCCTAGCTGAG	AGCTCAGTGAGGCTGATGT
RBX1	CACTAGGAAAAGACTTCTTCCATCAAG	TGACACAGAATACAATATGGCTACAGAAAC
RHOB	CTATGAGGGCCGCG	TAAAAAATAAAAAACAAGACAACAATTT

RNF6	TGGGTAAGGTGATGGGATCT	GGTAGAAACAGATCTTCAATGCATACTTT
RNFT1	TATTAAGTTGTATAAACTATCAAGGCCACAAAATA	CTGAAAACAATGTCAGGAAAGAATACC
SEMA6D	ACATGAATGTCCTCATCACCTG	ATTAATCTGTAATATTTTAGTTGCAAAGCTGA
SNAI1	CGCTGACCCTCGAG	GAATATCAATAAACTGTACATATAACTATA
SPRR1B	AAGTAATGTGGTCCACAGCC	GGGGGTATAAGGGAGCTG
SSBP2	GTGTGATCCATTACCAAGTCTC	GCTATGGTCTAAATGATTTGGGC
SUMO1	GTTTAGATATTCTTTTTATTTTTTTTCTTTTCCCTCAATC	CAACATGATTAGGTAACTGTATCATATATGCA
SUV39H2	AACTGAACTTTTTCAGGAAATAGAGCT	TTTATACCTTAAATTTCTTTATCATTGAGGTGCC
SYS1	GTCTAGAATCAGGCCCTTTG	CCTAAATCTTCATCCTGTCAG
TAPBPL	AGCTGACCTAAAGCGACATG	CTGAACAAAGGCAAAAAATACAAATT
TCEAL4	GTGTAGTGTCCCTGGCA	ATTTTTATGACTAAAGAAATCTGTTTTTAGGAG
TICAM2	GCCTGAGATGAAACATATAACATGTG	TTTAAGAAAGCCTGAGTAAGCATG
USP49	GGTTGATTTGTCCACATTTTATTGTTTTCCT	TATTTGAGTCAGGATCTCACTCTGTTG
WBP4	CAATAGTTGCAGGAGAGCTTTTTG	AAGCTTTTTTCTTACATGTACAGTCATCTTAA
WISP2	TTCTAGAGCCGGGCTG	CAGGAACAATTTTACAAACTCCAGA
XPA	ATGTGATTTTTTAGTTCAGTGACCTGT	TCTGCTATTAGGGCTTTTTCCAG
ZNF107	ACTTAATTGATCCTACAAGCTTACTACAC	ACATAAAAGTACAAATAGTAAAATGATATACTACTAATTCACTT
ZNF14	GTCTAAGAATATAAGCAACATTCTGAAGC	ACTGGGCTGTTTACATGATACTTT
ZNF800	GTCTGATAACTTCAAGTGATGTACGAAA	AAGACCTTGTGGATCTTTAACA

pBUTR - Site Directed Mutagenesis

**Gene**	**Forward primer (5'-3')**	**Reverse primer (5'-3')**
CRLF1_Mut.	ACCTTTGGGTGCACCCCAAGGGTTGGTTGAG	CTCAACCAACCCTTGGGGTGCACCCAAAGGT
DUSP2_Mut. 1	CTGGGGACTTGGGAAGAGCCTTTCACACCTGT	ACAGGTGTGAAAGGCTCTTCCCAAGTCCCCAG
DUSP2_Mut. 2	ACAACCAGGAGCCCTTCGTCTGCCCAGG	CCTGGGCAGACGAAGGGCTCCTGGTTGT
JUNB_Mut.	ACTTAGTCTCTAAAGAGTTTATTTTAAGACTTCTTTTTATTGAATCTATTTAGTGGGTTG	CAACCCACTAAATAGATTCAATAAAAAGAAGTCTTAAAATAAACTCTTTAGAGACTAAGT
SNAI1_Mut.	GATTCCTGAGCTGGCCATCCAGAGCTGTTTGG	CCAAACAGCTCTGGATGGCCAGCTCAGGAATC

qRT-PCR

Gene	Forward primer (5'-3')	Reverse primer (5'-3')
ACTB	ACCCAGCACAATGAAGATCA	ACATCTGCTGGAAGGTGGAC
ATF3	GTGTCCATCACAAAAGCCGA	AGGCACTCCGTCTTCTCCTT
CRLF1	CCCAGATCTCATAGGGCGTA	GAGACCTTCCTCCACACCAA
CYP4B1	ATCTACTGGCTCACCCCACA	ATGTCCAGGAAGTCCAGGTG
DUSP2	GCCTCCGCTGTTCTTCAC	AACCACTTTGAGGGCCTTTT
DYNLT3	TGGACTGCAAGCATAGTGGA	CTCTTCTGGACCACTGCACA
EGR3	GACAATCTGTACCCCGAGGA	TCCCAAGTAGGTCACGGTCT
EGR4	CAAAGCCCAGCTCAAGAAGT	TGCTCCACCTTAGCGAGTTT
FOSB	TCTGTCTTCGGTGGACTCCT	GAAGGAACCGGGCATTTC
GLIPR2	CACAATGAGTACCGGCAGAA	AGCCACCTCCTTTCCTGTCT
JUNB	AGCTACTCCCCAGCCTCTG	GGAGGTAGCTGATGGTGGTC
KLK7	TGCACGAAGGTTTACAAGGA	GGGTACCTCTGCACACCAAC
KRT4	ATTCTCACCTCGCTGCTCTG	TTGCAGAGCTCAACAGGATG
MALAT1	GAATTGCGTCATTTAAAGCCTAGTT	GTTTCATCCTACCACTCCCAATTAAT
MTRF1	GCACTGGAAGAAAGGCAAAC	GTCCTTCCAGCTGTCACCTC
NR4A1	TCCTGGAGCTCTTCATCCTC	GGCCAGGATACTGTCAATCC
NUF2	GAAAAACTTGCCACAGCACA	TCCCTTTCAGCAGCATCTTT
PADI2	GCGAATCACCATCAACAAGA	CTGTCAGTCCCAGCTCCTTC
PPARGC1A	GGCACGCAATCCTATTCATT	TGCCTGGAGACCTTGATCTT
RHOB	GAGAACATCCCCGAGAAGTG	CGAGGTAGTCGTAGGCTTGG
SEMA6D	ATGAGCCCTGGTTCACAAAG	AGGACTGGTCTTTGCCAGAA
SNAI1	GCGAGCTGCAGGACTCTAAT	GGACAGAGTCCCAGATGAGC
SSBP2	TAAGGGCACCATTCCTCTTG	CCCAGGAAGTCAGCCATTAC
TICAM2	CCCGGAATAATCTTTGCTGA	TCCATGCAGACCCATTTACA
WISP2	ATGAGAGGCACACCGAAGAC	AGGGGCAGGTACATGGTGTC

**RNA-IP/GRE Mutants**	
**Gene**	**Primer (5’-3’)**
tRFP_For	TGTCTTGTGCCCAGGAGAG
Renilla_For	ATTGAATCGGACCCAGGATTC
CRLF_Rev	CTCAGGTGCCCTGAAGTGAG
DUSP2_Rev	GGGCTTCTGAAACTCTGAGG
JUNB_Rev	GTAAACGTCGAGGTGGAAGG
SNAI1_Rev	ATTCCATGGCAGTGAGAAGG

**CELF1 –RNA binding mutants**		
**Gene**	**Forward primer (5’-3’)**	**Reverse primer (5’-3’)**
CELF1_G62C	ATCAAGATGTTTGTGGCCCAGGTTCCAAGGACC	GGTCCTTGGAACCTGGGCCACAAACATCTTGAT
CELF1_T181G	CCCGCCTCAGAGCAAAGGGGCCTGTTTTGTTACATTTTACA	TGTAAAATGTAACAAAACAGGCCCCTTTGCTCTGAGGCGGG
CELF1_G338C	GACAGGAAGCTGTTTATTGCTATGATTTCCAAGAAGTGC	GCACTTCTTGGAAATCATAGCAATAAACAGCTTCCTGTC
CELF1_T448G	CTGATGGCCTGAGCCGAGGTGCTGCATTTGTGACTT	AAGTCACAAATGCAGCACCTCGGCTCAGGCCATCAG
CELF1_G1322C	GGATTGTCGTAACTTACAAAAGCAAAACACTTGCTCAGGTTTG	CAAACCTGAGCAAGTGTTTTGCTTTTGTAAGTTACGACAATCC
CELF1_Frag1	AGGATGACGATGACAAGCTTATG	GGTCCTTGGAACCTGAGCCACAAACATCTTGAT
CELF1_Frag2	ATCAAGATGTTTGTGGCTCAGGTTCCAAGGACC	GTAAAATGTAACAAAACAGGCCCCTTTGCTCTGAGG
CELF1_Frag3	CCTCAGAGCAAAGGGGCCTGTTTTGTTACATTTTAC	CTTCTTGGAAATCATAGCAATAAACAGCTTCCT
CELF1_Frag4	AGGAAGCTGTTTATTGCTATGATTTCCAAGAAG	GTAAAAGTCACAAATGCAGCACCTCGGCTCAGGCC
CELF1_Frag5	GGCCTGAGCCGAGGTGCTGCATTTGTGACTTTTAC	GTCGTAACTTACAAAAGCAAAACACTTGCTCAG
CELF1_Frag6	CTGAGCAAGTGTTTTGCTTTTGTAAGTTACGAC	CTTCTGAGATGAGTTTTTGTTC

**Luciferase Constructs**		
**Gene**	**Forward primer (5’-3’)**	**Reverse primer (5’-3’)**
IRES	AGTCACGCTAGCGCCCCTCTCCCTCCC	TACTGTATTGCTCATATGGGTATTATCGTGTTTTTCAAAGGAA
CRLF1	TGGCTGTCTAGAGCTGTAGGGGCTCAGGCCA	AGGCTGGGGCCCTAAAAGGACTCTTTTGGAGGG
SNAI1	TGGCTGTCTAGACCCTCGAGGCTCCCTCTT	AGGCTGGGGCCCGAATATCAATAAACTGTACATATAACTATA
SSBP2	TGGCTGTCTAGAATCCATTACCAAGTCTCCTCATG	AGGCTGGGGCCCGCTATGGTCTAAATGATTTGGGC

Supplementary Table 2. List of *Silencer* Select and other siRNAs used in the current study. Note each gene was silenced with 2 different siRNAs. *NA*, not applicable.

**Gene**	***Silencer* Select siRNA 1 Catalog # or Sense Sequence 1 (5′–3′)**	***Silencer* Select siRNA 2 Catalog # or Sense Sequence 2 (5′–3′)**
CRLF1	s17673	s17674
CYP4B1	s3856	s3855
DUSP2	s4367	s4366
EGR3	s4545	s4544
FOSB	s230577	s223612
GLIPR2	s45646	s45644
JUNB	s7661	s7662
PADI2	s22189	s22187
PPARGC1A	s21393	s21394
RHOB	s1575	s1574
SEMA6D	s36854	s36853
SNAI1	s13186	s13187
SSBP2	s24244	s24245
TICAM2	s51478	s51476
CELF1	CCAUGAACGGCUUUCAAAUUGGAAU	GGACAGAUUGAAGAGUGCCGGAUAU
CELF2	CAGAGUAAAGGUUGUUGUUUCGUAA	GCUGGAGCCACUGUCGGAUUGAAUA
MBNL1	ACGACGUCAUUAGCCAUAUUGUAUA	CACAGCCAACCAGAUACCCAUAAUA
MBNL2	GAGAUUAAUGGGAGGAACAAUUUGA	GCGUUGCAUGAGGGAGAAAUGCAAA
Firefly Luciferase	GCACUCUGAUUGACAAAUACGAUUU	NA

Supplementary Table 3. List of antibodies used in the current study for immunoblot, immunoprecipitation (IP), immunohistochemistry (IHC), and flow cytometry. *NA*, not applicable (cases where the antibodies were used either for IP, IHC (concentrations are mentioned in relevant Methods section), or flow cytometry).

Name of Antibody	Vendor	Catalog #	Dilution
CELF1 (3B1)	EMD Millipore, Billerica, MA	05-621	1:1000
CELF1 (1.T.9)	Santa Cruz Biotechnology, Dallas, TX	56649	*NA* (IP)
CELF1 (3B1)	Abcam, Cambridge, MA	ab9549	*NA* (IHC)
CELF2 (1H2)	Kind gift from Dr. Thomas A Cooper	*NA*	1:1000
CRLF1	Sigma-Aldrich, St. Louis, MO	SAB2100484	1:1000
CYP4B1	ProteinTech, Chicago, IL	11771-1-AP	1:500
DUSP2	Thermo Scientific, Rockford, IL	PA5-28775	1:1000
E-cadherin (24E10)	Cell Signaling, Beverly, MA	3195	1:1000
E-cadherin APC conjugate (67A4)	BioLegend, San Diego, CA	324108	*NA* (Flow)
E-cadherin AF405 (67A4)	Santa Cruz Biotechnology, Dallas, TX	sc-21791 AF405	*NA* (Flow)
EGR3 (C-24)	Santa Cruz Biotechnology, Dallas, TX	sc-191	1:500
FIBRONECTIN (IST-9)	Abcam, Cambridge, MA	ab6328	1:1000
FOSB (H-237)	Santa Cruz Biotechnology, Dallas, TX	sc-28213	1:500
GLIPR2	Sigma-Aldrich, St. Louis, MO	HPA029478	1:250
JUNB (C37F9)	Cell Signaling, Beverly, MA	3753	1:1000
HSP90	BD Biosciences, San Jose, CA	610419	1:5000
MBNL1	LifeSpan Biosciences, Seattle, WA	LS-B4372	1:1000
MBNL2	Santa Cruz Biotechnology, Dallas, TX	Sc-136167	1:500
N-cadherin	Cell Signaling, Beverly, MA	4061	1:1000
PPARGC1A	Thermo Scientific, Rockford, IL	PA5-22958	1:1000
PADI2	ProteinTech, Chicago, IL	12110-1-AP	1:1000
RHOB (C-5)	Santa Cruz Biotechnology, Dallas, TX	sc-8048	1:500
SEMA6D	Sigma-Aldrich, St. Louis, MO	AV49583	1:1000
SNAI1	Bioss, Woburn, MA	bs-1371R	1:200
SSBP2	LifeSpan Biosciences, Seattle, WA	LS-B5585	1:500
TICAM2	Thermo Scientific, Rockford, IL	PA5-23396	1:250
VIMENTIN (RV202)	Abcam, Cambridge, MA	ab8978	1:1000
